# Becoming female: Ovarian differentiation from an evolutionary perspective

**DOI:** 10.3389/fcell.2022.944776

**Published:** 2022-09-07

**Authors:** Barbara Nicol, Martin A. Estermann, Humphrey H-C Yao, Namya Mellouk

**Affiliations:** ^1^ Reproductive and Developmental Biology Laboratory, National Institute of Environmental Health Sciences, Research Triangle Park, NC, United States; ^2^ Université Paris-Saclay, UVSQ, INRAE, BREED, Jouy en Josas, France

**Keywords:** chicken, mammals, granulosa cells, sex determination, ovary, teleost

## Abstract

Differentiation of the bipotential gonadal primordium into ovaries and testes is a common process among vertebrate species. While vertebrate ovaries eventually share the same functions of producing oocytes and estrogens, ovarian differentiation relies on different morphogenetic, cellular, and molecular cues depending on species. The aim of this review is to highlight the conserved and divergent features of ovarian differentiation through an evolutionary perspective. From teleosts to mammals, each clade or species has a different story to tell. For this purpose, this review focuses on three specific aspects of ovarian differentiation: ovarian morphogenesis, the evolution of the role of estrogens on ovarian differentiation and the molecular pathways involved in granulosa cell determination and maintenance.

## Introduction

In vertebrate species, ovaries arise from the gonadal primordium, a structure that has the bipotential capacity to differentiate into either ovaries or testes during embryogenesis. This dual developmental fate relies on the ability of gonadal cells to differentially respond to genetic or environmental cues that will dictate their fate toward ovarian or testis identity. Among the gonadal somatic cells, the supporting cells are considered the orchestrator of gonad differentiation. These supporting cells are usually the first gonadal cell-type to initiate their male or female fate and become Sertoli or granulosa cells respectively (Sinclair et al., 1990). For instance, in most mammals, the XY supporting cells first express the Y-linked sex-determining gene *SRY*, which activates the Sertoli-cell differentiation program that drives testis differentiation (Koopman et al., 1991; Albrecht and Eicher, 2001; Sekido and Lovell-Badge, 2008). In the absence of the Y-chromosome or of SRY, supporting cells differentiate into granulosa cells, tipping the gonadal fate toward ovaries. For this reason, ovarian differentiation has been considered a default process. However, genetic evidence from mice, humans, and other species have revealed that ovarian differentiation is in fact an active process that involves unique morphogenetic changes and activation/repression of specific genetic programs. Beyond mammals, bipotential gonads of all vertebrate species face this critical fate decision to differentiate into an ovary or a testis. Gonadal development and ovarian differentiation vary among vertebrate species. From different chromosomic systems (XX/XY vs. ZZ/ZW vs. polygenic), different sex determining triggers (*SRY* in mammals, *DMRT1/DMY* in chicken and medaka, *AMHY* in tilapia) (Nagahama et al., 2021), different cell origins, to divergent ovarian morphology, each clade or species adapts its unique molecular and cellular mechanisms. Despite this divergence, there is a large conservation of key genetic players and cell types involved in ovarian differentiation throughout vertebrate evolution.

In this review, we intend to generate a comparative view of ovarian differentiation from three main vertebrate clades: fish, birds, and mammals. We focus on three aspects of ovarian differentiation: *1*) ovarian morphogenesis and origins of granulosa cells; *2*) the evolutionary role of estrogens in ovarian differentiation; and *3*) the genetic pathways that regulate granulosa cell/ovarian differentiation.

## Ovary morphogenesis and origins of granulosa cells

In vertebrates, the ovary develops from the gonadal primordium that eventually form a structure composed of follicles, the functional units of the ovary. Follicles are composed of three key cell populations: an oocyte, surrounded by supporting granulosa cells, themselves surrounded by steroidogenic theca cells. Precursors of these cell populations are suspected to be present in the gonadal primordium. The primordium arises from the convergence of primordial germ cells, along with coordinated events of epithelial-mesenchymal transition (EMT) from the coelomic epithelium/lateral plate mesoderm and cell migration from the mesonephric/pronephric region. Morphogenesis of the ovary has been studied in various species and the origin of ovarian granulosa cells has been a long ongoing debate. It was already speculated in the 1960s/70s that mammalian granulosa cells could arise from either two waves of recruitment from the ovarian surface epithelium to form medullary and cortical cords; or from the mesenchyme; or even from cells of mesonephric origin, the “rete ovarii” (for review, *see* Byskov, 1986). While vertebrate ovaries eventually share the same functions of producing oocytes and reproductive hormones, the ovarian morphogenesis varies from species to species.

### Fish

Teleosts are the largest infraclass of the Actinopterygii, the ray-finned fishes. They present a variety of sex determination strategies that range from gonochorism, when the bipotential gonads directly differentiate into either an ovary or a testis, to sequential hermaphroditism, when a species switches sex later in life. Such diversity is caused by the capacity of the gonad to follow various genetic and/or environmental cues in a species-specific manner. Consequently, the timing and process of ovarian morphogenesis differ greatly from one fish species to another.

**FIGURE 1:**
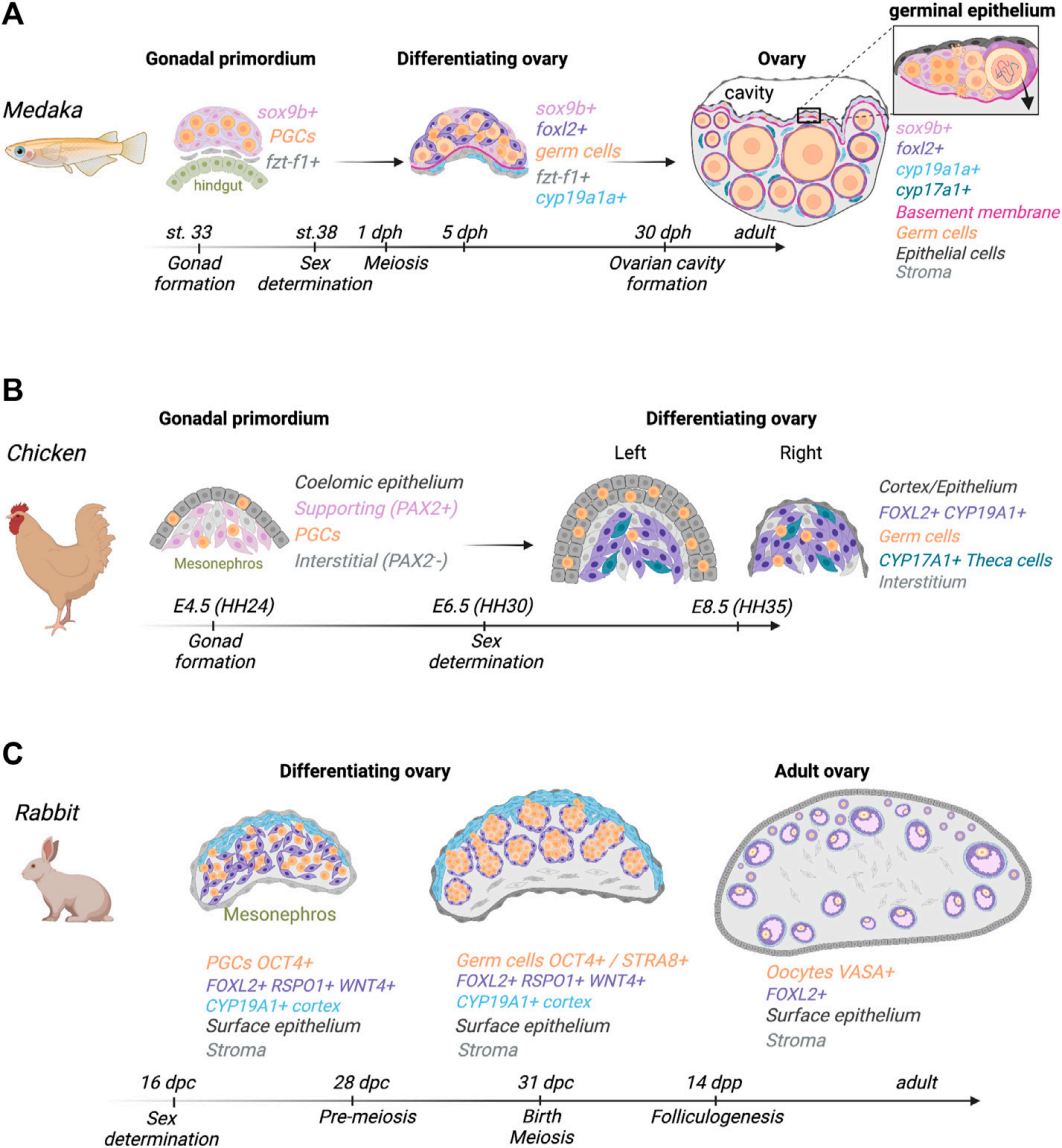
Comparative ovarian morphogenesis in medaka, chicken, and rabbit. **(A)** In medaka, the gonadal primordium is composed of three cell populations: primordial germ cells (PGCs), *ftz-f1+* cells, and *sox9b*+ supporting cell precursors. Sex determination is initiated just before hatching, at stage 38. *sox9b*+ cells surround PGCs to give rise to the germinal cradles that later form a multilayered germinal epithelium along with epithelial cells. At hatching, a basement membrane is formed at the ventral side. Meiosis is initiated 1-day post-hatching (dph), and *foxl2* becomes expressed in supporting cells surrounding diplotene oocytes that exit cradles. Around 5 dph, *cyp19a1+* becomes expressed in stroma cells in the ventral region. As follicles grow in the stroma, *foxl2*+ granulosa cells recruit *cyp19a1a*+ cells and *cyp17a1*+ theca cells. Ovarian cavity is formed around 30 dph. Adult ovaries still contain a germinal epithelium (inset) composed of undifferentiated *sox9*+ supporting cells, and germline stem cells that synchronously divide, enter meiosis and either die or exit the cradle to form follicles in the stroma. **(B)** In chickens, the gonadal primordium consists of two main compartments, the coelomic epithelium and the underlying mesenchyme. In the left ovary, the coelomic epithelium gives rise to the cortex and interstitial cells. *FOXL2* ^+^/*CYP19A1* ^+^ pre-granulosa cells and *CYP17A1*+ steroidogenic cells derive from a PAX2^+^ mesenchymal population. Germ cells that migrate into the gonad from the bloodstream accumulate in the cortical region of the left ovary. The right ovary does not form a cortex and germ cells remain in the medulla. **(C)** In rabbits, ovarian cell differentiation starts at 18 dpc and is associated with an upregulation of *FOXL2*, *WNT4* and *RSPO1* in somatic cells. At this stage, proliferating cells in the coelomic epithelium start to express *CYP19A1*. At 30 dpc, somatic cells expressing *FOXL2*, *WNT4* and *RSPO1* surround *OCT4/STRA8* positive germ cells and form ovigerous cords. The expression of *STRA8* signals meiosis initiation. At 14 dpp, the formation of the first primordial follicles is initiated. Then, growing follicles are composed of *FOXL2*+ granulosa cells enclosing *VASA*+ oocytes.

The medaka (*Oryzias latipes*) is one of the most-studied fish species regarding sex-determination and differentiation. It is a gonochoristic species, and the bipotential gonads differentiate into an ovary or a testis based on the presence of XX or XY sex chromosomes with the Y-linked sex determining gene *dmy/dmrt1by* (Matsuda et al., 2002; Nanda et al., 2002). Gonadal ridges form between the hindgut and nephric ducts around 3 days post-fertilization (dpf) (Figure 1A). Two somatic cell gonadal precursors originate from the lateral plate mesoderm: cells expressing *ftz-f1*, ortholog of *Nr5a1*, located laterally to the hindgut, and cells expressing *sox9b*, located more dorsally between the hindgut and nephric duct (Nakamura et al., 2006). The migrating primordial germ cells (PGCs), *sox9b*+ cells and *ftz-f1*+ cells meet dorsally around stage 33 (around 4.4 dpf) to form a single gonadal primordium (Figure 1A). The *sox9b+* cells correspond to supporting cell progenitors that surround germ cells to form bipotential “units” common to both male and female gonads (Nakamura et al., 2008; Nishimura et al., 2016). As *sox9b*+ cells surround PGCs, they start expressing *ftz-f1* (Nishimura and Tanaka, 2014). These *sox9b*+ bipotential supporting cells form interwoven threadlike cords that surround nests of germline stem cells to form germinal cradles. These cradles, along with epithelial cells, constitute the multilayered germinal epithelium that contribute to egg production throughout adult life (Nakamura et al., 2010). The first sexually dimorphic difference is the presence of more germ cells in female gonads than the male gonads 1 day before hatching at stage 38 (Satoh and Egami, 1972), followed by meiosis initiation 1-day post-hatching (dph) (Nakamura et al., 2006). After initiation of ovarian differentiation at hatching, diplotene oocytes individually surrounded by *sox9b+* supporting cells exit the cradles for the stroma. These supporting cells further differentiate into granulosa cells by downregulating *sox9b* expression and activating *foxl2* (Nakamoto et al., 2006). Meanwhile, around 5 dph, *cyp19a1a* becomes expressed in stroma cells located in the ventral region of the differentiating ovary (Figure 1A) (Nakamura et al., 2009). Once in the stroma compartment, granulosa cells recruit two types of steroidogenic stroma cells, *cyp19a1a*+ cells and *cyp17a1*+ cells, to form the outer theca layer of the follicles (Nakamura et al., 2009). As the gonad differentiates into an ovary, it is composed of three main structures: *1*) the stroma, located in the ventral side where follicles will grow; *2*) the germinal epithelium containing the germinal cradles; and *3*) the ovarian cavity, formed around 30 dph, into which mature oocytes are ovulated (Figure 1A).

On the other hand, the zebrafish (*Danio rerio*) has a different strategy of sex determination and gonad differentiation from the medaka. While the zebrafish is widely used as a model organism to study developmental processes, sex determination and gonadal differentiation are still poorly understood and varies between wild (ZZ/ZW system) vs laboratory (lack sex chromosomes) strains (for review, *see* Kossack and Draper, 2019). Zebrafish presents a juvenile hermaphroditism, as males and females first develop transient juvenile ovaries containing oocytes during larval life (∼13–25 dpf) (Takahashi, 1977). Gonadal development starts with the appearance of an elongated gonadal primordium around 5 dpf (Braat et al., 1999). Around 8–10 dpf, at least two populations of gonadal somatic cells co-exist: an outer layer of epithelial *fgf24*+ cells that become located to the dorsal edge of the gonad; and an inner layer of *fgf24*-/*etv4*+ cells in closer contact with germ cells and whose development appears to rely on the outer *fgf24*+ cell population (Leerberg et al., 2017). A subset of germ cells become oogonia and initiate meiosis in all gonads (Tong et al., 2010). At 12 dpf, the *fgf24*- cells start to separate into three distinct sub-populations: *etv4*+ cells, *cyp19*+ cells likely representing steroidogenic cell precursors (Dranow et al., 2016) and *amh*+ cells likely representing supporting cell precursors (Rodríguez-Marí et al., 2005). A few cells expressing *gsdf*, a marker specific of supporting cell lineage (Gautier et al., 2011; Yan et al., 2017), are detected as early as 8 dpf and appear to originate from the dorsal epithelium to eventually surrounds germ cells (Song et al., 2021). Gonadal differentiation is initiated around 20–25 dpf. The first clear morphological change between sexes is the degeneration of meiotic oocytes in presumptive males (Uchida et al., 2002). During this period (20–25 dpf), expression of the granulosa cell marker *wnt4a* is maintained in supporting cells of the presumptive ovary whereas it is lost in the presumptive testis (Kossack et al., 2019). On the other hand, the Sertoli-cell marker *sox9a* is lost in the presumptive ovary but maintained in the presumptive testis (Rodríguez-Marí et al., 2005). *cyp19a1a* only becomes expressed in granulosa cells later, when granulosa cells surround oocytes that are past mid-Stage II (Dranow et al., 2016). Similar to medaka and other fish species, zebrafish ovary contains a germinal epithelium at the surface of the ovary, composed of germline stem cell (GSC) surrounded by bipotential supporting cells (Beer and Draper, 2013), that contribute to continuous production of follicles throughout life, ovary regeneration and gonad plasticity (Cao et al., 2019).

### Avians

In birds, the gonadal primordium first appears in the ventromedial surface of the mesonephros around embryonic day 4.5 (E4.5) (Figure 1B) (Rodemer et al., 1986). Lineage tracing experiments in the chicken embryo evidenced that the coelomic epithelium contributes to the gonadal epithelial and interstitial cells, but not to the supporting cells, as opposed to mammals (Sekido and Lovell-Badge, 2007; Estermann et al., 2020). Instead, the supporting cell population derives from the mesonephric mesoderm, which itself is of intermediate mesodermal origin. More specifically, these supporting cells derive from a *PAX2/DMRT1/WNT4/OSR1* positive mesenchymal population (Figure 1B) (Estermann et al., 2020). It is worth noting that both *OSR1* and *PAX2* are some of the earliest intermediate mesoderm markers (James et al., 2006), supporting the intermediate mesoderm origin of avian supporting cells. Moreover, PAX2-positive progenitor cells were identified in the mesenchymal region of undifferentiated quail (Galloanserae), zebrafinch (Neoaves) and emu (Palaeognathe) gonads (Estermann et al., 2021b). This suggests that the mesenchymal origin of supporting cells is conserved among all bird clades.

Undifferentiated avian gonads exhibit a left/right asymmetry, impacting later ovarian development. This asymmetry is the result of increased proliferation in the left gonadal epithelium, rather than an increase in epithelial apoptosis in the right gonad (Ishimaru et al., 2008). *RALDH2*, the enzyme responsible for retinoic acid synthesis, is expressed asymmetrically in the right epithelium of the undifferentiated chicken gonads at E5-5.5. Retinoic acid suppresses ERα and *NR5A1* expression, resulting in the downregulation of cyclin D1, one of the main proliferation regulators. In the left gonad, the expression of *PITX2* inhibits *RALDH2* expression, upregulating NR5A1, ERα, cyclin D1 and consequently stimulating cell proliferation (Guioli and Lovell-Badge, 2007; Ishimaru et al., 2008; Rodríguez-León et al., 2008). Ovarian sex determination occurs at E6.5-E7. During ovarian differentiation, the left ovary eventually becomes enlarged and a thick multi-layered cortex forms, surrounding the underlying medulla (Figure 1B) (Guioli and Lovell-Badge, 2007). This becomes morphologically evident at E8.5. Estrogens, synthesized in the ovarian medulla, play a crucial role in the cortical formation through ERα signaling (Lambeth et al., 2013; Guioli et al., 2020). On the contrary, the epithelium of the right ovary does not form a cortex and regresses over time (Figure 1B). Despite lacking a cortex, the right gonad remains as a steroidogenic organ, being able to produce estrogens during embryonic development (Guioli et al., 2020). Most proliferating primordial germ cells (PGCs) in the developing left ovary are located in the cortical region (Figure 1B). Around E10.5, *RALDH2* starts expressing in the ovarian left cortex, whereas *CYP26B1* expression is restricted to the juxtacortical medulla (Smith et al., 2008a). This results in higher retinoic acid levels in the left cortex. Around E15.5, these germ cells enter meiosis and later arrest at prophase I. In the right gonad, the PGCs undergo some proliferation but do not enter meiosis and later die (Ukeshima, 1996). Development of the functional left ovary is completed after hatching with the formation of follicles in the cortex, harboring the oocytes.

The medullary region of the fetal ovary comprises three main cell populations: the *FOXL2*+/*CYP19A1*+ pre-granulosa cells, the steroidogenic theca cells, and the interstitial/stromal cells (Figure 1B) (Estermann et al., 2020). Between the cortex and the medulla of the left ovary, an accumulation of interstitial cells forms a compact structure called the juxtacortical medulla (Estermann et al., 2021a). This structure derives from the gonadal epithelium by EMT. The functional significance of this structure remains unclear, but it is the site of expression of the retinoic acid degrading enzyme CYP26B1 later in development and might be implicated in meiosis (Smith et al., 2008a).

After sex determination, pre-granulosa cells and germ cells are located into two distinctive compartments in the ovary. Germ cells are in the cortical and/or juxtacortical medulla (JCM) region, whereas the pre-granulosa cells are in the medulla. Granulosa and germ cells must associate to form the ovarian follicles. In E14.5 chicken ovaries, *FOXL2*+/ERα- cells accumulate in the cortical or juxtacortical medulla region of the ovary (Major et al., 2019). The origin of these cells is currently unknown. One of the possibilities is that the medullary pre-granulosa cells migrate into the JCM/cortical region, guided by epithelial or oocyte-secreted factors. On the other hand, these *FOXL2*+ cells could derive from cortical cells through EMT, downregulate ERα and upregulate *FOXL2* along the process. If that is the case, the medullary pre-granulosa cells could function as a source of steroids due to the presence of androgenic theca cells and estrogenic pre-granulosa cells. It is unclear if *CYP19A1* is expressed in these cortical/juxtacortical *FOXL2*+ cells.

### Mammals

#### Mouse

The development of the mouse ovary starts during the first half of fetal development at E9.5-11. Similar to other mammal species, bipotential gonad emerges from the thickening of the coelomic epithelium of the intermediate mesoderm (Moritz and Wintour, 1999; Bunce et al., 2021). The bipotential gonad is composed of primordial germ cells and somatic precursor cells, including supporting cells and interstitial cells that can follow either a testis or an ovary fate (Stevant et al., 2019). In the XX gonad, supporting cells are responsible for the initiation of ovarian determination by differentiating into pre-granulosa cells. Pre-granulosa cells arise from two timely waves of differentiation (Mork et al., 2012; Zheng et al., 2014; Niu and Spradling, 2020). The first wave of pre-granulosa cells arises from the *Runx1*+ bipotential supporting cells. As sex determination is initiated, *Runx1* expression is maintained in XX gonads whereas repressed in XY gonads (Nicol et al., 2019; Stevant et al., 2019). These *Runx1*+ supporting cells start expressing *Foxl2* around E12 and give rise to medullary pre-granulosa cells. Starting around E12.5, a second wave of pre-granulosa cell population arises from *Lgr5*+ cells of the ovarian surface epithelium that ingress into the ovary (Gustin et al., 2016; Niu and Spradling, 2020; Fukuda et al., 2021). These cells give rise to cortical pre-granulosa cells, which eventually lose *Lgr5* expression and upregulate *Foxl2* shortly after birth (Rastetter et al., 2014; Feng et al., 2016; Stevant et al., 2019; Cai et al., 2020; Niu and Spradling, 2020). The medullary granulosa cells are responsible for the first wave of folliculogenesis after puberty, whereas the cortical granulosa cells are involved in the second wave of folliculogenesis during the adult life (Mork et al., 2012; Zheng et al., 2014; Niu and Spradling, 2020). Primordial germ cells (PGCs) reach the gonad between E10 and 11.5 days, when sex determination of the somatic cells is initiated. The commitment of PGCs into male or female gametes depends on their somatic environment. The female germ cells proliferate until they initiate meiosis. Meiosis begins at E12.5 in a few cysts in the antero-medial region and radiates outward (Soygur et al., 2021), followed by an anterior to posterior initiation wave at E13.5 (Menke et al., 2003). At this stage, female PGCs are surrounded by a layer of pre-granulosa cells to form ovigerous cords. Around birth, granulosa cells break down the ovigerous cords by enclosing single oocytes, leading to the formation of primordial follicles. Finally, theca cells are recruited postnatally from both the ovarian stroma and the mesonephros to surround developing follicles (Liu et al., 2015).

#### Rabbit

In rabbits, the gestation lasts 31 days and sex determination happens at 16 dpc. The bipotential gonads appear around 14 dpc (Mario et al., 2018) (Figure 1C). The first event of somatic cell differentiation starts at 16 dpc (Mario et al., 2018) and is associated with a regression of the mesonephros between 16 dpc to 25 dpc (Bernier and Beaumont, 1964). Ovarian commitment is initiated at 18 dpc with the expression of *FOXL2*, *RSPO1* and *WNT4* (Daniel-Carlier et al., 2013). At 23 dpc, the gonadal and mesonephric tissues are separated by connective tissue that is supposed to prevent the migration of cells and other substances (Hayashi et al., 2000). At 30 dpc, the ovigerous cords and the surrounding stromal tissue are compactly arranged in the ovarian cortex, whereas in the medullary region, the stromal tissue is loosely arranged, and the ovigerous cords are easily discerned and maintain continuity with the surface epithelium (Diaz-Hernandez et al., 2019). In parallel, the germ cells express the pre-meiotic marker STRA8 and enter a pre-meiotic phase from 24 to 28 dpc. Then, meiosis occurs asynchronously during the 15 days following birth and germ cells remain arrested in prophase I of meiosis. At this stage, the rupture of the ovigerous nests followed by the formation of the first primordial follicles is initiated at the interface between the cortex and medulla of the ovary.

#### Ruminants

In farm animal species, ovaries can be identified at 20–23 dpc in sheep, 25 dpc in goat and 32 dpc in cow (Mauleon, 1976; McNatty et al., 1995; Pailhoux et al., 2002). Before sex determination, cells from the mesonephros migrate and populate the gonadal primordium (Zamboni et al., 1979; Pailhoux et al., 2002; Kenngott et al., 2013). In cows, cells from the surface epithelium, named gonadal ridge epithelial-like cells (GREL) give rise to pre-granulosa cells (Hummitzsch et al., 2013). A similar cell population is suspected to exist in the sheep ovary (Juengel et al., 2002). Primordial germ cells are observed at the genital ridge at 25 dpc in goat (Pailhoux et al., 2002) from 21 dpc in sheep (Ledda et al., 2010) and 31 dpc in cow (Wrobel and Suss, 1998). Ovarian morphological differentiation and cortical development is first apparent at 29 dpc in sheep, 34–36 dpc in goat and 42 dpc in cow. Proliferating germ cells and pre-granulosa form cord-like structures named ovigerous nests (Juengel et al., 2002; Pannetier et al., 2012; Hummitzsch et al., 2013). Around 55 dpc in goat and sheep and 75 dpc in cow, female germ cells enter meiosis while mesonephric-derived somatic cells colonize the genital ridge. Contrary to mice, primordial follicles form during gestation, around 75 dpc in sheep, 90 dpc in goat and 130 dpc in cow (Erickson, 1966; Pailhoux et al., 2002; Sawyer et al., 2002).

#### Human

The human gonadal primordium first becomes discernible around 4 weeks post-conception (wpc), and PGCs reach the genital ridges between 4 and 6 wpc. The gonadal primordium is composed of a proliferating coelomic epithelium and an underlying compartment containing mesenchymal cells, blood vessels and mesonephric cells (Byskov, 1986). Sex determination is initiated just before 6 wpc, when XY gonads start expressing *SRY* in the supporting cell precursors (Mamsen et al., 2017). In the ovary, the cortex further develops, and poorly defined ovarian cords start to form around 8 wpc with connections to the ovarian surface epithelium. These cords are composed of germ cell clusters surrounded by a single layer of flattened supporting cells. Human granulosa cells are suspected to arise, at least in part, from the surface epithelium, similar to findings from the mouse, cow, and sheep. Indeed, electron microscopy revealed that the extension of human ovarian cords correlates with ingrowths of proliferating surface epithelium (Motta and Makabe, 1982). Around 11 wpc, some germ cells initiate meiosis (Gondos et al., 1986). Following meiosis entry, waves of germ cell apoptosis occur throughout the rest of gestation. Germ cell cyst breakdown starts at mid-gestation, around 20 wpc in the human ovary.

### Evolutionary perspectives

From fish to human, ovaries share the same purpose of producing oocytes and reproductive hormones. However, some differences during their morphogenesis leads to species-specific properties. A major difference during vertebrate evolution is the retention of germline stem cell (GSC) surrounded by bipotential supporting cells in fish. This germinal epithelium has the capacity to produce unlimited oocytes throughout life and to regenerate the ovary. It is also likely responsible for gonadal plasticity leading to fully functional sex reversal in adult fish upon various genetic or environmental cues. Birds and mammals have lost this capacity to regenerate the ovary. Instead of a germinal epithelium, they form an ovarian cortex containing the stock of follicles that will be used later in life.

Ovarian morphogenesis is conserved among mammals, with the formation of ovigerous cords that eventually break down to form follicles. A key difference among mammals is the timing of meiosis initiation and follicle formation. Meiosis is only initiated after birth in rabbit, contrary to mice and other mammals. Similarly, follicle formation happens postnatally in mouse and rabbit and during gestation in larger mammals. The evolutionary and functional meaning of these differences may be related to the extension of gestation period in larger mammals.

In all clades, ovaries are composed of three main cell populations: germ cells, supporting cells and mesenchymal/steroidogenic cells. It has become clear that in mammals, granulosa cells arise at least in part from the ovarian surface epithelium. On the other hand, in chicken, granulosa cells derive from mesonephric mesoderm. Lineage tracing experiments helped tackling the question of origins of somatic cell populations. Unfortunately, it remains difficult to compare these findings between species as genes used for cell lineage tracing, such as *fgf24* or *gsdf*, were lost during vertebrate evolution (Jovelin et al., 2010; Hsu and Chung, 2021). With more scRNA-seq datasets becoming available for differentiating ovaries from various species, it would provide the opportunity to not only compare the cell populations identified, but an evolutionary analysis of gonadal cell lineages. Such comparisons will help decipher convergent and divergent origins of ovarian cell populations among vertebrates.

Finally, most birds present an intriguing feature during ovarian morphogenesis: they only keep one functional ovary. In chickens, despite developing both left and right gonads during early embryogenesis, only the left one remains as a fully functional ovary whereas the right ovary regresses. It is still unknown what the evolutionary mechanism is for developing one main ovary. Fossilized remains of birds from the early Cretaceous period suggest this trait was acquired early in birds’ evolution (Zheng et al., 2013).

## Role of estrogens in ovarian differentiation

The role of estrogens in ovarian differentiation has been studied for many decades in various vertebrate species. Estrogens represent the main hormones produced by the ovary. They are produced through the conversion of androgens by the enzyme CYP19A1, also known as aromatase. By binding to their receptors (ERα and β), estrogens regulate gene expression and thus ovarian differentiation and/or development. The use of aromatase inhibitors, exogenous estrogens and transgenic models were key to elucidate the role of estrogens in ovarian differentiation in a wide range of organisms.

### Fish

**FIGURE 2:**
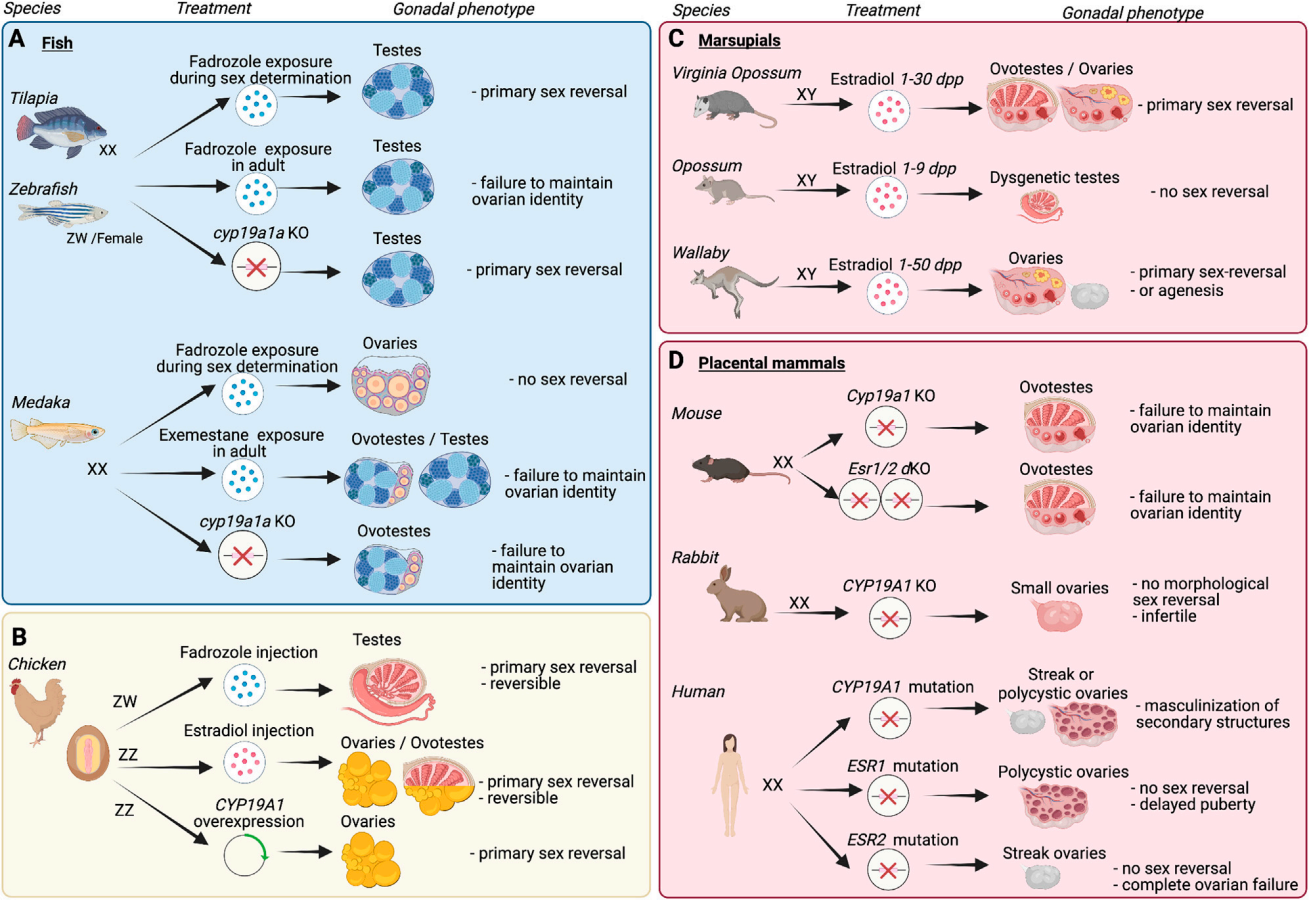
Effects of manipulating estrogen signaling on the gonadal fate in fish, chickens, and mammals. **(A)** *In fish*: In female tilapia and zebrafish, both exposure to aromatase inhibitor fadrozole during sex determination and knockout of *cyp19a1a* cause primary ovary-to-testis sex reversal, while long-term fadrozole treatment of mature females leads to ovary-to-testis sex reversal. Therefore, *cyp19a1a* is involved in both primary ovarian differentiation and maintenance of ovarian identity. In medaka, exposure to fadrozole during sex determination has no effect on ovarian differentiation, while both *cyp19a1a* KO and treatment of adult females with aromatase inhibitor exemestane eventually masculinize the ovaries. This indicates that *cyp19a1a* is only involved in the maintenance of medaka ovarian identity. **(B)** *In chickens*: treatment with aromatase inhibitor fadrozole in ZW female embryos results in transient testis development. On the other hand, estradiol injection to ZZ male embryos results in ovary or ovotestis differentiation. Moreover, ovarian development is induced in male embryos overexpressing CYP19A1. **(C**,**D)** *In mammals*: **(C)** *In marsupials*. Effects of estradiol treatment on XY males varies from species to species. Newborn XY Virginia opossum exposed to estradiol for 30 days post-partum (dpp) develops ovaries or ovotestes, whereas newborn XY gray short-tailed opossum exposed for 9 dpp develops dysgenetic testes. Newborn XY wallaby exposed for 50 dpp develops ovaries or gonadal agenesis if born prematurely. **(D)** *In placental mammals:* In the mouse, *Cyp19a1* knockout or *Esr1/Esr2* double knockout leads to postnatal transdifferentiation of granulosa cells into Sertoli-like cells. This indicates a role in the maintenance of ovarian identity rather than in primary ovarian differentiation. In the rabbit, *CYP19A1* knockout does not impair primary ovarian differentiation but prevents the proper development of ovaries, resulting in small ovaries and infertility. In humans, mutations in *CYP19A1* or *ESR1* or *ESR2* genes do not impact primary ovarian differentiation but cause either streak ovaries or polycystic ovaries.

In many fish species, manipulations of estrogen signaling at specific windows of development lead to fully functional sex-reversal (for review, *see* Guiguen et al., 2010; Li et al., 2019) (Figure 2A). With the teleost-specific whole genome duplication (Meyer and Van de Peer, 2005), *cyp19a1* is present in two sub-functionalized copies: *cyp19a1a* is specifically expressed in the ovary, while *cyp19a1b* is expressed in the brain and occasionally in the ovary (Chang et al., 2005). *cyp19a1a* is expressed before histological differentiation of the gonads in various fish species (Vizziano et al., 2007; Dranow et al., 2016). Teleosts have at least three estrogen receptors (*esr1*, *esr2a* and *esr2b*) that are expressed in both male and female developing gonads, explaining their sensitivity to the feminizing effects of estrogens.

In tilapia, treatment of XX larvae with the non-steroidal aromatase inhibitor fadrozole during sex determination causes masculinization of the gonads (Kwon et al., 2000). Meanwhile, long-term treatment of adult females with fadrozole transforms the ovaries into functional testes (Paul-Prasanth et al., 2013; Sun et al., 2014). Furthermore, *cyp19a1a* knockout causes primary ovary-to-testis sex reversal, with upregulation of Sertoli gene *dmrt1* at the time of sex determination (Zhang et al., 2017). The transcription factor Dmrt1 binds *cyp19a1a* promoter and represses its expression (Wang et al., 2010). These observations support the role of *cyp19a1a* and estrogens in both primary ovarian determination and maintenance of ovarian identity in adulthood. Loss of either of the three estrogen receptors does not lead to masculinization, suggesting some functional redundancy between estrogen receptors (Yan et al., 2019).

In zebrafish, exposure to fadrozole during sex determination causes testis differentiation (Fenske and Segner, 2004). Loss of *cyp19a1a* also leads to testis development (Lau et al., 2016; Yin et al., 2017). This sex-reversal phenotype in the *cyp19a1a* knockout model is rescued by additional loss of *dmrt1*, suggesting that estrogen signaling controls ovarian differentiation by repressing *dmrt1* in zebrafish (Wu et al., 2020). Long-term treatment of mature females with fadrozole results in ovary-to-testis sex-reversal, indicating that *cyp19a1a* is also required for the maintenance of ovarian identity (Takatsu et al., 2013). Similar to tilapia, none of the three estrogen receptors single KO results in ovary-to-testis sex-reversal (Lu et al., 2017). Nonetheless, in the absence of all three receptors, ovaries eventually transdifferentiate into ovo-testes or testes. While the triple estrogen receptor KO leads to an all-male phenotype, it does not fully recapitulate the *cyp19a1* KO phenotype, which prevents primary ovarian differentiation. It remains unclear how estrogens drive early ovarian differentiation independently of estrogen receptors in zebrafish.

In medaka, brief exposure to estrogens just 1 day post-fertilization causes functional testis-to-ovary sex reversal, similar to other fish species (Kobayashi and Iwamatsu, 2005). Yet, intrinsic estrogen production is not critical for primary ovarian differentiation. Indeed, *cyp19a1a* expression only appears in the ovarian interstitium several days after sex determination is initiated (Suzuki et al., 2004). Exposure to fadrozole during gonad differentiation after hatching does not affect ovarian development (Suzuki et al., 2004). Meanwhile, long-term exposure to the aromatase inhibitor exemestane in adult females causes functional ovary-to-testis sex reversal (Paul-Prasanth et al., 2013). Loss of *cyp19a1* in medaka does not impair initial ovarian differentiation, but later leads to ovary degeneration and partial ovary-to-testis sex reversal after puberty (Nakamoto et al., 2018). Therefore, in medaka, endogenous estrogens are only necessary for the maintenance of ovarian identity. Similar to tilapia and zebrafish, none of the three estrogen receptors single KO cause ovary-to-testis sex reversal (Kayo et al., 2019), but the effects of combined loss remain to be determined.

### Avians

In avian species like chicken, endogenous estrogens have a central role in ovarian development (Smith et al., 1997; Brunström et al., 2009). Unlike mammals, the estrogen-synthesizing enzyme CYP19A1 is expressed in female gonads at the onset of ovarian differentiation (Figure 1B) (Elbrecht and Smith, 1992; Andrews et al., 1997; Nomura et al., 1999). This results in elevated ovarian and systemic estrogen levels in females from the time of sex determination (George and Wilson, 1982; Tanabe et al., 1986; Elbrecht and Smith, 1992). *CYP19A1* is detected in pre-granulosa cells and in a subset of embryonic theca cells (Estermann et al., 2020). Steroidogenic theca cells are responsible for the synthesis of androgens, which then are converted into estrogens by CYP19A1. Chicken embryonic ovaries contain more *CYP17A1*+ steroidogenic cells than testes (Estermann et al., 2020). This likely reflects the higher demand of androgens in the ovary to be converted into estrogens. In chickens, embryonic steroidogenic theca cells were found to derive from the female supporting cell lineage. A subset of *FOXL2* ^+^/*CYP19A1* ^+^ pre-granulosa cells in the medulla upregulates the androgen-producing enzyme *CYP17A1* and become transitioning/intermediate cells, expressing both theca and granulosa cell markers (Estermann et al., 2020). Then, pre-granulosa cell markers are downregulated whereas other steroidogenic markers are upregulated, completing the differentiation towards theca cells (Estermann et al., 2020). It is still unknown if these embryonic theca cells become the adult theca cells, or if they have additional origins as in mice (Liu et al., 2015).

Estrogen is required for ovarian cortex formation and pre-granulosa differentiation. In the left chicken ovary, the epithelium becomes multilayered, forming the ovarian cortex that harbors meiotic germ cells. The chromosomal composition (ZZ or ZW) does not contribute to cortex formation (Guioli et al., 2020). Instead, estrogens, synthesized locally by CYP19A1 in the medulla, are required to induce cortex formation through ERα signaling. When ERα is knocked down, the cortex fails to form in the ovary (Elbrecht and Smith, 1992; Guioli et al., 2020).

Treatment with aromatase inhibitors fadrozole or letrozole in ZW chicken embryos inhibits estrogen synthesis, upregulate DMRT1, resulting in ovary-to-testis sex reversal at E6.5 (Figure 2B) (Smith et al., 2003; Trukhina et al., 2014). This sex reversal is not permanent as ZW embryos treated with fadrozole at E3.5 eventually upregulate *CYP19A1* and revert to ovotestis, (Figure 2B) (Nishikimi et al., 2000; Vaillant et al., 2001; Estermann et al., 2021a). Conversely, exposure of genetically male (ZZ) chicken embryos to exogenous 17β-estradiol results in ovarian development, that reverses back to testis upon estradiol decay (Figure 2B) (Bannister et al., 2011; Guioli et al., 2020; Shioda et al., 2021). Moreover, constitutive *CYP19A1* over-expression in ZZ embryos leads to complete ovary formation (Figure 2B) (Lambeth et al., 2016). Altogether, these experiments implicate the formative role of estrogen in inducing the program for ovary formation.

### Mammals

#### Marsupials

Marsupials constitute a clade of non-placental mammalians that includes opossums, wallabies, kangaroos, koalas, wombats, Tasmanian devils, and bandicoots. Marsupial embryonic development is characterized by a premature birth. These immature newborns crawl up into their mothers’ pouch (marsupium), attach themselves to a teat, and continue their development (Mahadevaiah et al., 2020; Smith and Keyte, 2020). Gonadal sex determination in marsupials occurs after birth, while they are growing in the pouch (Renfree and Short, 1988; Baker et al., 1993; Renfree et al., 1996). This external development makes them a remarkable model to study embryonic gonadal differentiation, compared to the *in-utero* development of eutherian mammals.

Although estrogens are not synthesized in early embryonic ovaries, marsupial sex determination is sensitive to exogenous estrogens (George et al., 1985; Renfree et al., 1992) (Figure 2C). This could be attributed to the expression of both estrogen receptors α and β in undifferentiated gonads and in differentiated supporting and germ cells (Calatayud et al., 2010). *In vitro* and *in vivo* analysis in different marsupial species demonstrate that estrogens have a role in inhibiting testicular development or inducing ovarian development. In the Virginia opossum (*Didelphis virginiana*), exposure of XY embryos to estradiol dipropionate for 30 days post-partum (or dpp) leads to formation ovotestes or a complete testis-to-ovary sex reversal (Figure 2C) (Burns, 1955). In the gray short-tailed opossum (*Monodelphis domestica)*, estradiol benzoate treatment to XY embryos from days 1–9 post-partum result in dysgenetic testes, but not testis-to-ovary sex reversal (Figure 2C) (Fadem and Tesoriero, 1986). In the tammar wallaby (*Macropus eugenii*), estradiol benzoate treatment to XY embryos at birth for 25 days result in ovotestis development at day 25 post-partum (Shaw et al., 1988), followed by complete testis-to-ovary sex reversal at 50 dpp (Coveney et al., 2001) (Figure 2C). In prematurely born XY embryos, estradiol benzoate treatment caused gonadal agenesis. *In vitro* culture of tammar wallaby gonads was used to study the molecular mechanisms responsible of estrogen mediated gonadal sex reversal (Calatayud et al., 2010; Pask et al., 2010). In cultured XY gonads, estrogens reduce *SRY* and *AMH* expression and induce *FOXL2* and *WNT4* expression. While *SOX9* expression was not downregulated, estrogen treatment inhibits SOX9 translocation into the nucleus, consequently preventing activation of the testis differentiation pathway (Pask et al., 2010).

#### Mouse

In the mouse, neither estrogen receptors (*Esr1/2*) nor *Cyp19a1* are expressed in the fetal ovary during sex determination at E10-11.5. ERα (encoded by *Esr1*) and ERβ (encoded by *Esr2*) become detected in the fetal ovary later on, around E14.5-E15.5 (Lemmen et al., 1999; Chen et al., 2009). In the neonatal ovary, ERα is expressed in somatic cells and ERβ is detected in oocytes (Chen et al., 2009). In the adult ovary, ERα is mainly expressed in theca cells and ERβ in granulosa cells (Jefferson et al., 2000). There are some discrepancies regarding whether *Cyp19a1* is expressed in the fetal ovary after sex determination. Some studies show that *Cyp19a1* expression is only detected close to birth (Greco and Payne, 1994) whereas others detect CYP19A1 protein as early as E13.5 (Dutta et al., 2014). Inactivation of *Cyp19a1* does not impact early ovarian differentiation but the females are infertile (Fisher et al., 1998) (Figure 2D). Further analyses revealed that *Cyp19a1* KO mouse ovaries present some testis-like structures with Sertoli-like cells after postnatal follicle formation, and this masculinization could be reversed by exposure to estrogens (Britt et al., 2001; Britt et al., 2002; Britt et al., 2004). Similarly, while single loss of *Esr1* or *Esr2* does not impact ovarian identity, their combined loss causes postnatal transdifferentiation of granulosa cells into SOX9+ Sertoli-like cells (Couse et al., 1999). Estrogen receptors cooperate with FOXL2 to maintain the identity of ovarian granulosa cells through repression of SOX9 (Uhlenhaut et al., 2009; Georges et al., 2014). In addition, FOXL2 ChIP-seq revealed that *Esr2* is a direct target of FOXL2 in adult granulosa cells, and that FOXL2 positively regulates *Esr2* (Georges et al., 2014). Altogether, these results indicate that estrogen signaling is not necessary for ovarian determination but is required for the maintenance of granulosa cell identity in mice.

#### Rabbit

In rabbits, CYP19A1 expression in the ovary and the capacity of fetal and neonatal granulosa cells to synthesize steroid hormones has been well described (Gondos, 1969; Erickson et al., 1974; George and Wilson, 1980; Gondos et al., 1983). *CYP19A1* expression starts at 16 dpc and reaches its peak at 20 dpc. At this stage, *CYP19A1*, *ESR1* and *FOXL2* are located in distinct cell populations. CYP19A1 and ESR1 are expressed in the coelomic epithelium and contribute to cortex development whereas FOXL2 is expressed in the supporting cells located in the medulla (Jolivet et al., 2022). Concomitantly, intracytoplasmic lipid droplets, which provide the main source of cholesterol for steroid synthesis, accumulate in granulosa cells at 19 dpc (Gondos et al., 1983). Based on these observations, it has been hypothesized that this estrogen surge plays a role during rabbit ovary differentiation. However, loss of *CYP19A1* in the XX rabbit embryo has no impact on fetal ovary formation (Figure 2D), although ovaries are smaller and eventually contain a reduced follicular reserve (Jolivet et al., 2022). In the *CYP19A1* KO adult ovary, DMRT1 expression is upregulated in some granulosa cells while SOX9 expression is absent, and no structural masculinization of the ovary is observed. Estrogens appear to be dispensable for ovary formation in XX rabbit, but they play an important role in the establishment of the ovarian reserve by maintaining granulosa cell and germ cell proliferation.

#### Human

Transcriptomic analyses of human gonads during sex determination showed that both *ESR1* and *ESR2* are expressed in the gonads (Mamsen et al., 2017; Lecluze et al., 2020). There are however discrepancies whether their expression is stronger in the ovary than in the testis (Lecluze et al., 2020) or not different between sexes (Mamsen et al., 2017). During the second trimester, CYP19A1 protein is sporadically detected in the fetal ovary at 12 weeks of gestation and becomes strongly expressed by 19 weeks. CYP19A1 is detected in somatic cells surrounding oocyte nests and in granulosa cells of primordial follicles in the fetal ovary (Fowler et al., 2011). Patients with mutations in *CYP19A1* gene do not develop ovary-to-testis sex reversal (Figure 2D), but present ambiguous external genitalia at birth (Shozu et al., 1991; Mazen et al., 2017; Hathi et al., 2022). These patients tend to have either streak or polycystic ovaries (Mazen et al., 2017; Praveen et al., 2020). *ESR1* or *ESR2* mutations do not cause ovary-to-testis sex reversal or ambiguous external genitalia at birth, but ovaries present a polycystic or streak phenotype respectively (Quaynor et al., 2013; Bernard et al., 2017; Lang-Muritano et al., 2018). Such difference in ovarian phenotype suggests a more prominent role for *ESR2* than for *ESR1* during early ovarian development. Overall, these studies indicate that endogenous estrogens and their actions are not required for ovarian determination in humans, but mis-regulation of estrogen signaling has dramatic effects on ovarian development/function and physiology in adulthood.

### Evolutionary perspectives

From fish to human, estrogen signaling appears to be less and less involved in primary ovarian determination but remains critical for proper ovarian development and functions. In eutherian mammals, sex determination became de-coupled from the feminizing effects of estrogens as found in fish, birds, and other vertebrate species. This could be an evolutionary response to the maternal estrogens that pass through the placenta. In marsupials, gonadal sex determination occurs after birth, not being influenced by the maternal estrogens. Despite this, they are susceptible to external estrogens, a potential link to a more ancestral state that was lost in eutherian mammal evolution.

Estrogen receptors are expressed in ovarian embryonic somatic cells of mice, humans, goats, sheep, and marsupials, indicating that regulation of some elements of the estrogen signaling machinery remain conserved throughout vertebrate ovary differentiation. One key difference arises from the timing and location of *CYP19A1* expression. In fish and chicken, *CYP19A1* is not exclusively expressed in granulosa cells, but is detected in some stromal steroidogenic cells during early ovarian differentiation. These non-granulosa CYP19A1+ cells are not found in mammalian embryonic ovaries. This raises the question whether this unique population is responsible for estrogens action in ovarian determination, and the loss of this cell population contributed to the decoupling of estrogen involvement in ovarian determination in mammals. In both chicken and rabbit, estrogen signaling is required for cortex development in the developing ovary, contributing to cell proliferation and expansion of the cortex. It would be interesting to determine if this function is also conserved in other vertebrate species.

Altogether, these findings provide a potential explanation for the major shift in vertebrate sex determination mechanisms from an estrogen dependence on ovarian differentiation to a strictly genetic regulation.

## Genes and pathways controlling granulosa cell differentiation and ovarian identity

During sex differentiation, the gonad develops into an ovary or a testis, depending on the signaling pathways activated or repressed in the supporting cells. While the master sex determining gene varies among vertebrate species, the genes or pathways involved in pre-granulosa and ovarian differentiation are relatively conserved. *FOXL2* is one of the most conserved pro-ovarian genes in vertebrates and beyond. Independent of *FOXL2*, the WNT4/β-catenin pathway is also important for ovarian development in vertebrate species. Genome editing technologies such as CRISPR/Cas9 enabled the generation of knockout models in more and more non-model vertebrate species. Comparison of knockout models for these main pathways in various species helps determining the evolution of the role of these pathways during vertebrate ovarian differentiation. In addition, in recent years, omics technologies led to the identification of novel candidate genes involved in ovarian differentiation. The following sections compare and contrast the genes and pathways that control granulosa cell differentiation and ovarian identity.

### Fish

Compared to tetrapods, teleosts generally have two copies of each gene due to the teleost-specific whole genome duplication (Meyer and Van de Peer, 2005). This potentially results in sub-functionalization or neo-functionalization of genes and impacts genes associated with ovarian differentiation.

**FIGURE 3:**
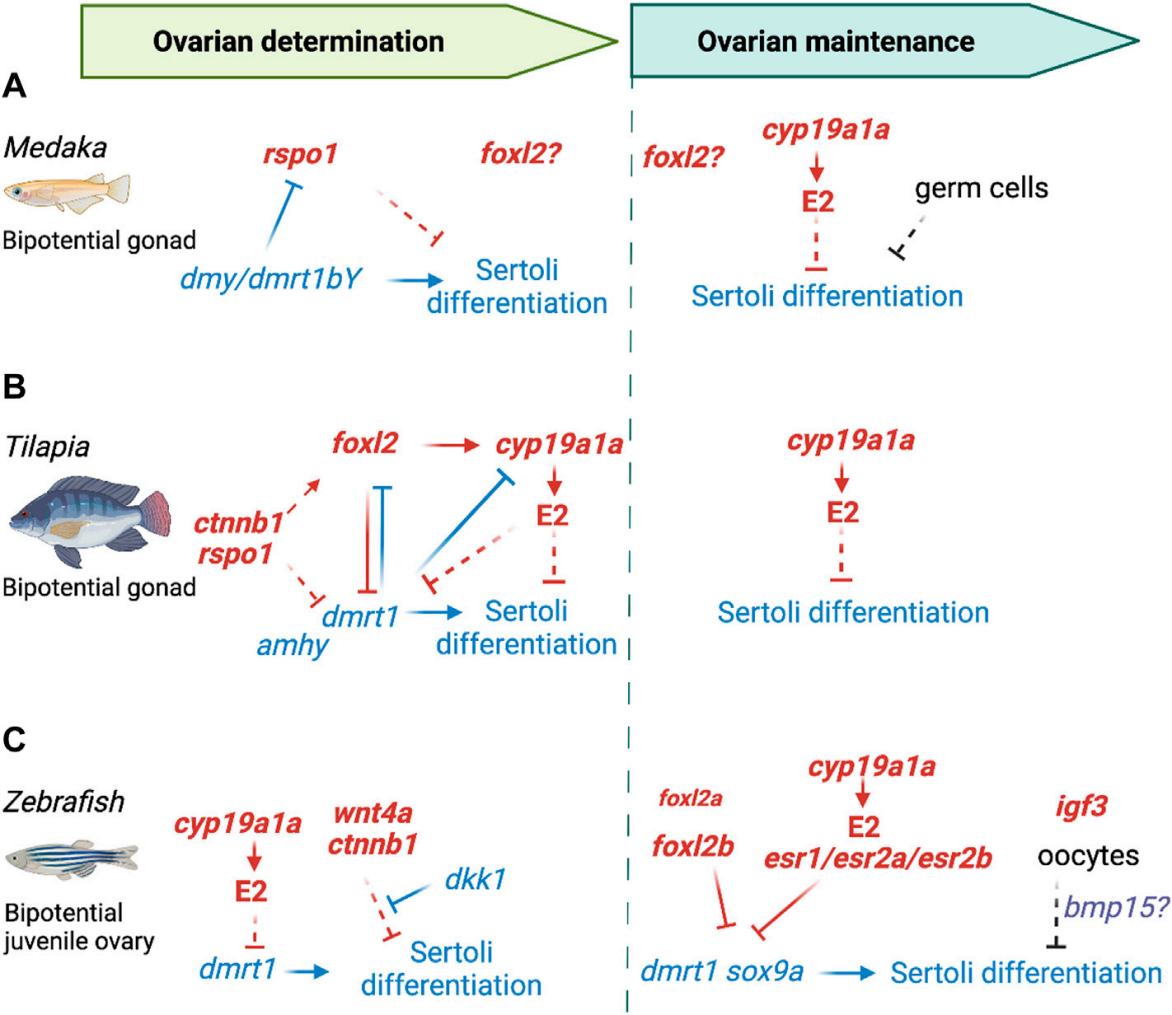
Genetic regulation of pre-granulosa cell differentiation in fish. **(A)** In medaka, in the absence of the Y-linked sex determining gene *dmy*, pro-ovarian genes (*rspo1* and later *foxl2*) are upregulated. *rspo1*, which is directly repressed by Dmy, is sufficient to fully drive ovarian differentiation in XY gonads. The functional role of *foxl2* remains to be determined. *cyp19a1* is only involved in the maintenance of ovarian differentiation. Loss of germ cells prevents the maintenance of granulosa cell fate. **(B)** In tilapia, in the absence of the Y-linked sex determining gene *amhy*, pro-ovarian genes are quickly upregulated. Repression of Wnt/β-Catenin pathway through either *rspo1* KO or exposure to inhibitors leads to repression of *foxl2* and upregulation of *dmrt1*, causing Sertoli cell differentiation. Loss of *foxl2* results in absence of *cyp19a1* expression and upregulation of *dmrt1* at time of sex determination. There is mutual antagonism between pro-testis gene *dmrt1* and pro-ovary genes *foxl2/cyp19a1*. *cyp19a1* is also involved in the maintenance of ovarian identity. **(C)** In zebrafish, all larvae first develop a bipotential juvenile ovary. Loss of *cyp19a1a* leads to upregulation of *dmrt1* and Sertoli cell differentiation. Repression of the Wnt/β-Catenin pathway, through either *wnt4a* KO or overexpression of inhibitor Dkk1 causes Sertoli cell differentiation. Combined loss of either *foxl2a/foxl2b*, or the three estrogen receptors impairs the maintenance of granulosa cell identity, resulting in complete ovary-to-testis sex reversal. Oocytes are required for the maintenance of granulosa cell fate. Loss of genes involved in PGC/oocyte development, such as *igf3*, results in ovary-to-testis sex-reversal. Bmp15 is suspected to be the oocyte secreted factor that directly acts on supporting cells.

*foxl2* gonadal expression has been analyzed in more than two dozen fish species, revealing a higher expression in the embryonic ovaries vs testes in almost all species studied (for review, *see* Bertho et al., 2016). In medaka (Figure 3A), *foxl2* expression is initiated after ovarian differentiation has started (Nakamoto et al., 2006). *foxl2* functions in medaka ovarian differentiation remain to be determined. Foxl2 protein is detected in the nuclei of granulosa cells, as well as in a subpopulation of Cyp19a1a+ stromal/theca cells (Nakamoto et al., 2006; Herpin et al., 2013). This expression of *foxl2* in some steroidogenic cells has been observed in other fish species such as tilapia (Wang et al., 2007) and gibel carp (Gan et al., 2021). In tilapia (Figure 3B), loss of *foxl2* causes complete ovary-to-testis sex-reversal (Li et al., 2013; Zhang et al., 2017). The role of the transcription factor Foxl2 in fish ovarian differentiation seems to rely on its capacity to directly induce *cyp19a1a* expression (Wang et al., 2007; Yamaguchi et al., 2007; Bertho et al., 2018; Gan et al., 2021; Yan et al., 2021), and/or to repress the pro-Sertoli gene *dmrt1* (Fan et al., 2019). Loss of *foxl2* in tilapia results in absence of *cyp19a1* expression and upregulation of *dmrt1* at time of sex determination (Zhang et al., 2017). In zebrafish (Figure 3C), *foxl2a* and *foxl2b* are both expressed in granulosa cells, but they present some sub-functionalization in the ovary (Yang et al., 2017). Single loss of *foxl2a* or *foxl2b* does not impair initial ovarian differentiation, but respectively leads to premature ovarian failure or partial sex-reversal in adult females. Their combined loss causes upregulation of Sertoli genes *dmrt1* and *sox9a* and complete ovary-to-testis sex reversal weeks after gonad differentiation. This phenotype suggests a cooperative role of these two sub-functionalized *foxl2* variants in maintaining ovarian identity in zebrafish, with a predominant role by *foxl2b*.

The role of the Rspo1/Wnt**/**β**-**catenin pathway in ovarian differentiation has been investigated in various fish species. R-spondin 1 (Rspo1) is a secreted factor that potentiates the canonical Wnt**/**β**-**catenin pathway. Expression of *rspo1* is higher in the differentiating ovary than in the testis in several fish species (Zhang et al., 2011; Zhou et al., 2012; Liu et al., 2018). In medaka, *rspo1* is detected at the onset of sex determination, around hatching, earlier than *foxl2*. At 10 dph, *rspo1* is detected in ovarian germ cells and surrounding somatic cells (Zhou et al., 2012). Ectopic expression of *rspo1* in XY medaka causes complete testis-to-ovary sex reversal and the development of fertile females (Zhou et al., 2016). Medaka Y-linked sex determining factor Dmy/Dmrt1bY, and its homolog Dmrt1a, both bind to *rspo1* promoter and repress its expression *in vitro* (Zhou et al., 2016). In the Nile tilapia (*Oreochromis niloticus)*, a gonochoristic species with a XX/XY sex determination system (Li et al., 2015), *rspo1* is expressed in germ cells in both sexes and becomes more expressed in the ovary before meiosis initiation. Reduction of *rspo1* expression causes both defects in ovarian and testis development in tilapia (Wu et al., 2016). Beside *rspo1*, other members of the Wnt**/**β**-**catenin pathway are more expressed in the differentiating ovary. In medaka, both *wnt4b* and *ctnnb1* present a sexually dimorphic expression (Zhou et al., 2016). In zebrafish, *wnt4a* becomes specifically expressed in somatic cells surrounding larger oocytes. Both *wnt4a* KO (Kossack et al., 2019) and Wnt antagonist *dkk1* overexpression cause testis development (Sreenivasan et al., 2014), confirming a key role of the Wnt**/**β**-**catenin pathway in ovarian differentiation in zebrafish. Different chemical inhibitors of the Wnt**/**β**-**catenin pathway have been used in other fish species, resulting in downregulation of pro-ovarian genes such as *cyp19a1a* or *foxl2* in tongue sole (Zhu et al., 2017) and carp (Wu et al., 2019) or upregulation of pro-testis gene *dmrt1* in tilapia (Wu et al., 2016). This suggests a conservation of the Wnt**/**β**-**catenin pathway functions in ovarian differentiation in teleosts, although each actor of the pathway may not play the same part, depending on the species. This also raises the question whether other Wnt ligands could be involved in ovarian differentiation.

Granulosa cell differentiation or the maintenance of their identity is also influenced by signals from the germ cells in fish. Beyond their capacity to intrinsically determine their female fate through *foxl2l* (formerly *foxl3*) (Nishimura et al., 2015; Dai et al., 2021), fish germ cells have a feminizing effect on the surrounding somatic environment. While loss of germ cells has no impact on ovarian differentiation in mice (Maatouk et al., 2012), absence of germ cells causes gonad masculinization in medaka (Kurokawa et al., 2007; Tanaka et al., 2008; Nishimura et al., 2018), zebrafish (Slanchev and Stebler, 2005; Siegfried and Nüsslein-Volhard, 2008) and hermaphroditic rice field eel (Hou et al., 2022). In XX medaka, in the absence of germ cells, supporting cells initiate their differentiation into granulosa cells, but they eventually start expressing Sertoli-specific genes (Kurokawa et al., 2007; Nishimura et al., 2018). Therefore, germ cells are required for the maintenance of granulosa cell differentiation rather than their initial determination. It remains unclear how germ cells influence their somatic environment. In zebrafish, whose gonads first form transient juvenile ovaries, it is hypothesized that oocytes secrete factors that prevent somatic cell masculinization. Dimorphic proliferation of PGCs is observed at 14 dpf, a week before gonad differentiation (Tzung et al., 2015). Reduction in germ cell number, whether it happens at larval or adult stage, causes ovary-to-testis sex reversal (Dranow et al., 2013; Dai et al., 2015; Tzung et al., 2015). In zebrafish, multiple ovary-to-testis sex reversal phenotypes are indirectly caused by the knockout of genes involved in PGCs migration, such as *adamts9* or *prmt5* (Carter et al., 2019; Zhu et al., 2019; Carver et al., 2021), in oocyte development, such as *bmp15* and *gdf9* (Dranow et al., 2016; Chen et al., 2017), *nobox* (Qin et al., 2022), *igf3* (Xie et al., 2021), RNA-binding proteins *rbpms2a/b* and *ddx5* (Kaufman et al., 2018; Sone et al., 2020), or genes from the Fanconi Anemia/BRCA DNA repair pathway (Rodríguez-Marí et al., 2010; Ramanagoudr-Bhojappa et al., 2018). Among them, Bmp15 is suspected to be the oocyte secreted factor that directly acts on supporting cells through binding to its receptor Bmpr2a/b at their surface (Dranow et al., 2016). Finally, loss of *nr0b1* (or *dax1*) leads to a skewed sex-ratio toward male, likely as a result of a significant decrease in germ cell numbers, although a role directly on somatic cells cannot be excluded (Chen et al., 2016).

### Avians

**FIGURE 4:**
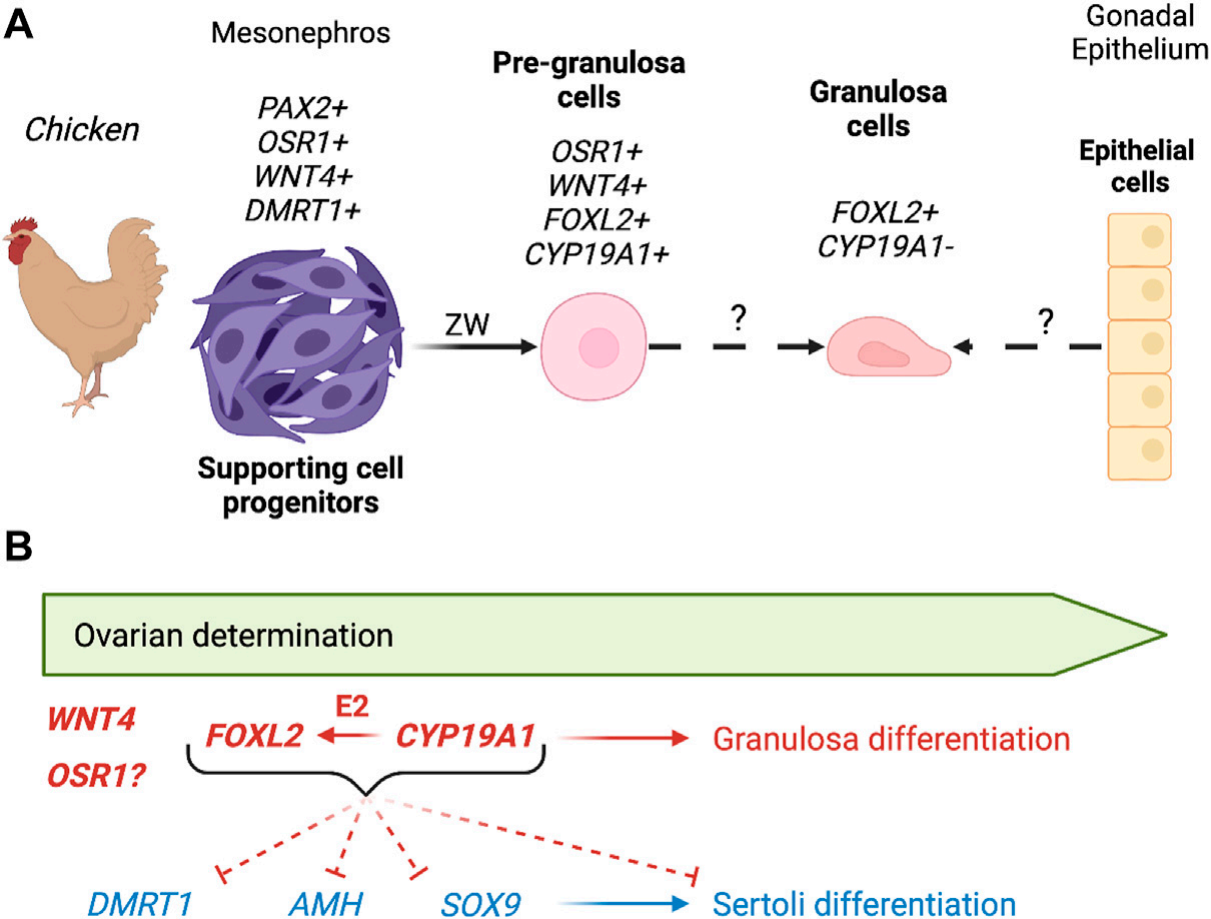
Genetic regulation of pre-granulosa cell differentiation in birds. **(A)** In chickens, the pre-granulosa cells derive from a *PAX2*/*OSR1*/*WNT4*/*DMRT1* positive mesenchymal cell population. During sex differentiation, they downregulate *DMRT1* and *PAX2* and start upregulating key pre-granulosa cell genes *CYP19A1* and *FOXL2*. It is still unknown if these medullary pre-granulosa cells give rise to the granulosa cells in the adult ovary follicles. **(B)** Although *WNT4* and *OSR1* were identified as pre-granulosa cell genes, their functions have not been analyzed yet. *FOXL2* and *CYP19A1* downregulate Sertoli cell genes *DMRT1*, *AMH* and *SOX9*, inhibiting Sertoli cell differentiation and inducing pre-granulosa cell program. CYP19A1 is also known to regulate *FOXL2* expression. Red: pro-ovarian genes/pathways; Blue: pro-testis genes/pathways; Plain arrow: direct effect; Dotted arrow: indirect effect.

In chicken gonads, undifferentiated male and female supporting cells have similar transcriptomes to the differentiated pre-granulosa cells (Estermann et al., 2020), suggesting that progenitors of the supporting cells are primed to a ovarian (pre-granulosa) fate. Pre-granulosa showed a continuous differentiation process from the undifferentiated supporting cells at E4.5 to differentiated pre-granulosa cells at E10.5 (Estermann et al., 2020). On the contrary, Sertoli cell differentiation is characterized by a rapid transcriptional change in male, resulting in a clear lineage separation. A similar phenomenon was also described in mice (Stevant et al., 2019). As mentioned before, chicken undifferentiated supporting cells express four key markers, *PAX2*, *OSR1*, *WNT4* and *DMRT1* (Estermann et al., 2020) (Figure 4A). PAX2 expression is downregulated at the onset of sex differentiation in both males and females (E6.5) (Estermann et al., 2020; Estermann et al., 2021b), a pattern also identified in other bird species, including emu, quail, and zebra finch, precisely predicting the onset of sex determination in all the analyzed avian species (Estermann et al., 2021b).

*DMRT1* is the Z-linked testis-determining gene in birds (Smith et al., 2009; Lambeth et al., 2014; Ioannidis et al., 2021; Lee et al., 2021). Due to the lack of chromosome dosage compensation in birds, *DMRT1* is expressed twice as high in males (ZZ) than females (ZW). The higher level of *DMRT1* triggers the supporting cell differentiation into Sertoli cells by upregulating Sertoli markers *AMH* and *SOX9* and downregulating *WNT4* and *OSR1* and *PAX2* (Lambeth et al., 2014; Estermann et al., 2020). In females, a single dose of *DMRT1* is not sufficient to trigger Sertoli differentiation (Ioannidis et al., 2021), which leads to maintenance of pre-granulosa cell genes such as *WNT4* and *OSR1*, the downregulation of the undifferentiated marker *PAX2*, and upregulation of key granulosa genes *FOXL2* and *CYP19A1* (Figure 4A) (Major et al., 2019; Estermann et al., 2020). *FOXL2* is the first to be activated in pre-granulosa cells upon sex determination in chickens (Loffler et al., 2003; Major et al., 2019). *FOXL2* overexpression in male chicken gonads results in downregulation of *DMRT1*, *SOX9* and *AMH* (Figure 4B) (Major et al., 2019). Conversely, *FOXL2* knockdown in females results in upregulation of *SOX9*. Surprisingly, and unlike several fish species, *CYP19A1* expression is not altered by *FOXL2* knockdown or over-expression in chicken. *CYP19A1* overexpression in male chicken embryos induces ovarian differentiation, inhibiting *DMRT1*, *SOX9* and *AMH* and upregulating *FOXL2* (Figure 4B) (Lambeth et al., 2013). This demonstrates the importance of estrogens during ovarian sex determination. It is still unclear if estrogens synthesized by CYP19A1 activate FOXL2 gene expression through ERα signaling or if it’s an indirect effect of the downregulation of repressive male genes (Figure 4B).

In chickens, *WNT4* is expressed in undifferentiated supporting cells, but is downregulated in males after the onset of sex determination (Smith et al., 2008b). Both β-catenin and RSPO1 are expressed in the cortical region of the ovary (Smith et al., 2008b; Ayers et al., 2013). The role of these genes in the chicken ovary determination has not been explored but based on their expression pattern they might play a role in development of ovarian cortex.

*Odd-Skipped Related Transcription Factor 1* (*OSR1*) was not previously associated with mammalian sex determination or supporting cell differentiation. *OSR1* is expressed in differentiated chicken pre-granulosa cells, colocalizing with FOXL2 (Estermann et al., 2020). *OSR1* is also significantly enriched in Muscovy duck embryonic ovaries (Bai et al., 2020), and in snapping turtle (*Chelydra serpentina*) embryonic gonads at the female-producing temperature (Rhen et al., 2021). In *Xenopus* and zebrafish, *Osr1* was shown to control kidney development, but its contribution to gonadal development remains unexplored (Tena et al., 2007). In mice, lineage tracing experiments show that all gonadal somatic cells derive from a *Osr1*+ intermediate mesoderm/lateral plate mesoderm population from E8.5 to E9.5 (Sasaki et al., 2021). Most of the *Osr1* ^−/−^ mice die at early embryonic stages (E11.5-E12.5), and they lack intermediate mesoderm derivatives such as adrenal glands, metanephros and gonads (Wang et al., 2005). Therefore, *Osr1* is required for gonad formation in mice, but it remains unclear whether *Osr1* plays a role in ovarian differentiation.

### Mammals

#### Mouse

Advances in transcriptomics technologies enabled the identification of genetic programs controlling ovarian differentiation, from microarrays on purified cell populations (Nef et al., 2005; Bouma et al., 2010; Jameson et al., 2012), to bulk RNA-seq (Zhao et al., 2018), and now single-cell RNA-seq (Stevant et al., 2019; Niu and Spradling, 2020). At time of sex determination, supporting cell precursors are already primed toward granulosa cell fate (Jameson et al., 2012), similar to findings in chicken (Estermann et al., 2020). This female bias was further confirmed by enrichment in open chromatin near granulosa-promoting genes in both XX and XY E10.5 supporting progenitors (Garcia-Moreno et al., 2019). As gonads differentiate into ovaries, a robust female genetic program is established through timely activation or maintenance of pro-ovarian genes and repression of pro-testis genes (Nef et al., 2005; Jameson et al., 2012; Munger et al., 2013). This differentiation is associated with an increase in active enhancers located near granulosa-promoting genes. These enhancers are enriched for TCF and FOX binding motifs, suggesting activity of the WNT/β-catenin and FOXL2 respectively (Garcia-Moreno et al., 2019).

Before sex determination, *Rspo1* and *Wnt4* are expressed similarly in XX and XY supporting cell precursors (Stevant et al., 2019), and the canonical β-catenin pathway is active in the coelomic epithelium (Usongo and Farookhi, 2012). *Wnt4* and *Rspo1* are required for the proliferation of supporting cell precursors in both XY and XX gonads (Chassot et al., 2012). Then, as sex determination is initiated, *Wnt4* and *Rspo1* expression and cortical β-catenin pathway activity are only maintained in XX gonads (Vainio et al., 1999; Parma et al., 2006; Usongo and Farookhi, 2012). Deletion of any of the RSPO1/WNT/β-catenin pathway factors *Wnt4, Rspo1*, *Ctnnb1*, or RSPO1 putative receptor *Lgr4* results in partial ovary-to-testis sex reversal. These knockout models share similar phenotypes, with appearance of Sertoli-like cells, ectopic steroidogenic cells, and testis-specific coelomic vessel (Vainio et al., 1999; Chassot et al., 2008; Manuylov et al., 2008; Liu et al., 2009; Koizumi et al., 2015). A mutually antagonistic relationship exists between RSPO1/WNT/β-catenin pathway and pro-Sertoli genes such as *Sox9* and *Fgf9* (Kim et al., 2006; Lavery et al., 2012; Nicol and Yao, 2015; Tang et al., 2020). In absence of *Wnt4*, granulosa cells exit mitotic arrest, prematurely differentiate, and eventually transdifferentiate into Sox9+ Sertoli-like cells in the perinatal ovary (Maatouk et al., 2013). Stabilization of β-catenin in XX *Rspo1* KO ovary rescues the ovary-to-testis sex reversal (Chassot et al., 2008), confirming that *Rspo1* acts through the WNT/β-catenin pathway. Overexpression of β-Catenin in XY mice is sufficient to induce testis-to-ovary sex reversal (Maatouk et al., 2008). However, this is not the case when *Wnt4* or *Rspo1* are overexpressed in XY gonads, suggesting that additional factors are missing to efficiently stabilize β-Catenin signaling and drive granulosa cell differentiation in the XY gonads (Jordan et al., 2003; Buscara et al., 2009).

FOXL2 is a conserved transcription factor expressed in pre-granulosa cells from E12 to mature granulosa cells in adult ovaries (Schmidt et al., 2004). In the absence of *Foxl2*, XX embryos develop morphologically normal ovaries; then, granulosa cells gradually transdifferentiate into DMRT1+ Sertoli cells postnatally (Schmidt et al., 2004; Uda et al., 2004; Ottolenghi et al., 2005; Garcia-Ortiz et al., 2009; Nicol et al., 2019). In addition, conditional deletion of *Foxl2* in adult ovarian granulosa cells induces a reprogramming into Sertoli-like cells, appearance of Leydig-like cells and testosterone production (Uhlenhaut et al., 2009). Therefore, while FOXL2 is not necessary for the initiation of the pre-granulosa cell program, it plays an important role in maintaining its identity. FOXL2 cooperates with Estrogen receptors to maintain granulosa cell identity in adult ovaries. Indeed, a similar transdifferentiation phenotype is observed in adult XX mice lacking both *Esr1*/*Esr2* (Couse et al., 1999), or lacking both *Esr1* and one *Foxl2* allele in granulosa cells (Uhlenhaut et al., 2009). FOXL2 directly controls *Esr2* expression and cooperates with estrogen receptors to repress *Sox9* transcription in adult granulosa cells (Uhlenhaut et al., 2009; Georges et al., 2014). On the other hand, ectopic expression of *Foxl2* in XY somatic cells causes partial testis-to-ovary sex reversal (Ottolenghi et al., 2007; Garcia-Ortiz et al., 2009; Nicol et al., 2018). FOXL2 plays complementary roles with the WNT/β-catenin pathway during ovarian differentiation. Indeed, *Wnt4/Foxl2* and *Rspo1/Foxl2* compound knockout XX mice develop a more pronounced ovary-to-testis sex reversal phenotype than *Wnt4*, *Rspo1* or *Foxl2* single knockout XX mice (Ottolenghi et al., 2005; Auguste et al., 2011).

Additional genes are involved in ovarian differentiation, such as the transcription factors FOG2 and GATA4 (Manuylov et al., 2008). The identification of RUNX1, a transcription factor involved in cell-fate determination, further highlights the complexity of the ovarian pathway. RUNX1 is expressed in the bipotential supporting cell lineage and then detected in granulosa cells and ovarian surface epithelium (Nef et al., 2005; Nicol et al., 2019). RUNX1 plays redundant roles with FOXL2 during ovarian differentiation. Ablation of *Runx1* alone in somatic cells impacts common sets of genes without affecting granulosa cell identity in the fetal ovary. *Runx1/Foxl2* double knockout causes masculinization of the supporting cells in the fetal ovary (Nicol et al., 2019). RUNX1 chromatin occupancy partially overlaps with FOXL2, suggesting that RUNX1 and FOXL2 share common direct target genes during ovarian differentiation.

Overall, these models reveal the multilayered regulations of ovarian differentiation, with synergistic and complementary roles between RSPO1/WNT/β-catenin, FOG2/GATA4, FOXL2 and RUNX1 to drive and maintain granulosa cell identity.

#### Rabbit

**FIGURE 5:**
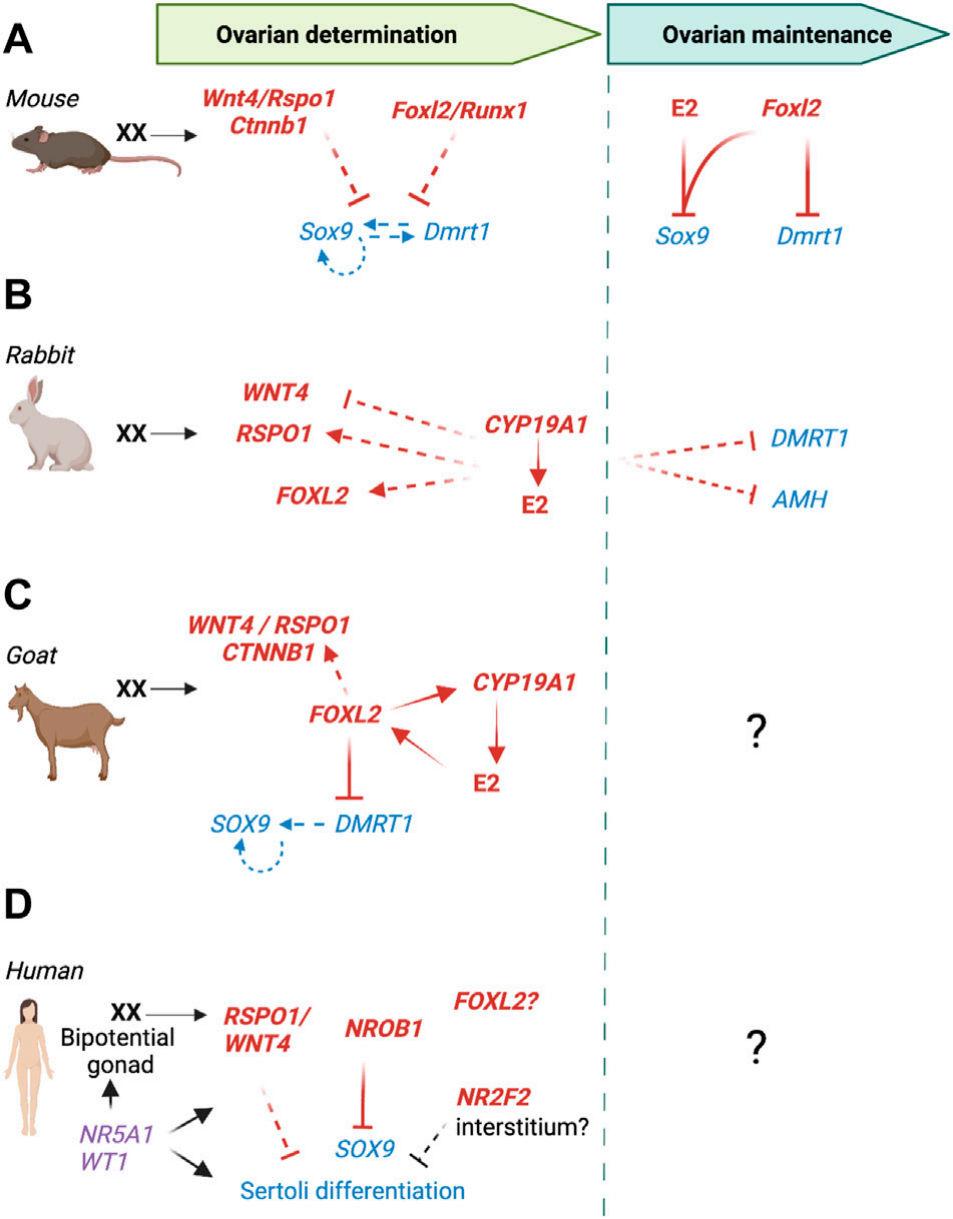
Genetic regulation of pre-granulosa cell differentiation in mammals. **(A)** In the mouse, RSPO1/WNT4/β-catenin pathway and *Foxl2/Runx1* tilt the balance towards the female side by silencing the expression of *Sox9*, *Dmrt1*, and downstream pro-Sertoli genes. Mutation in RSPO1/WNT4/β-catenin pathway or *Foxl2* causes partial ovary-to-testis sex reversal, whereas the combined loss of *Foxl2* with either *Runx1* or *Wnt4* or *Rspo1* leads to a more severe ovary-to-testis sex reversal. FOXL2 is also essential for the maintenance of granulosa cell identity in adult ovaries. The joint action of FOXL2 and estrogens (E2) enables the maintenance of ovarian identity. Loss of *Foxl2* or estrogen receptors *Esr1*/*Esr2* leads to an upregulation of SOX9 and DMRT1, and transdifferentiation of granulosa into Sertoli-like cells. **(B)** In the rabbit, *CYP19A1* is not involved in primary sex determination, but plays a crucial role in early ovarian development. Loss of *CYP19A1* reduces the expression of *FOXL2* and *RSPO1* in fetal ovaries and upregulates the expression of DMRT1 and AMH in some adult granulosa cells from large antral follicles, leading to ovarian defects without any sign of morphological gonad masculinization. **(C)** In the goat, FOXL2 is a crucial factor in early ovarian differentiation. FOXL2 directly controls *CYP19A1* *FOXL2* loss of function causes downregulation of the WNT/β-catenin pathway, upregulation of *DMRT1* followed by *SOX9*, leading to complete ovary-to-testis sex reversal. **(D)** In humans, *RSPO1* is one of the earliest sexually dimorphic pro-ovarian genes during gonad differentiation. Mutations in *WNT4* or *RSPO1* causes XX ovotesticular development while duplication of the genomic region containing both *WNT4* and *RSPO1* causes XY gonadal dysgenesis. Duplication of *NR0B1* (*DAX1*) causes XY male-to-female sex reversal with gonad dysgenesis. Mutations of *NR5A1* or *WT1* cause both XX and XY DSDs, likely due to their role in bipotential gonad formation. Mutation or deletion of a portion of *NR2F2* causes XX testis development. As *NR2F2* expression is detected in interstitium, it is unclear how this gene impacts granulosa cell identity. It remains unclear whether FOXL2 is involved in ovarian determination as heterozygous mutations cause folliculogenesis defects rather than gonad masculinization. Red: pro-ovarian genes/pathways; Blue: pro-testis genes/pathways; Plain arrow: direct effect; Dotted arrow: indirect effect.

In the rabbit gonads, expression of *FOXL2*, *RSPO1* and *WNT4* marks the commitment toward an ovarian fate (Daniel-Carlier et al., 2013). *WNT4* and *RSPO1* are expressed in both XX and XY undifferentiated gonads. As sex determination is initiated at 16 dpc, *WNT4* and *RSPO1* are upregulated in XX gonads, reaching a peak of expression at 24 dpc and 7 dpp respectively, to eventually decrease to minimal levels in adult ovaries. *FOXL2* expression is first detected between 16 and 18 dpc and rises gradually to reach a maximum that is sustained in adults (Daniel-Carlier et al., 2013). FOXL2 protein is detected in granulosa cells at 18 dpp and RSPO1 in the germ cell cytoplasm in the cortical zone at 4 dpp. To date, no knockout models are available to study the role of the *WNT4*, *RSPO1* and *FOXL2* in the rabbit ovary. In the XX *CYP19A1* KO fetal ovary, the expression of both *FOXL2* and *RSPO1* is decreased at 22 dpc while WNT4 expression is increased, suggesting that WNT/β-catenin signaling is stimulated in KO ovaries and may contribute to absence of sex reversal (Figure 5B) (Jolivet et al., 2022).

#### Goat

In goats, a naturally occurring deletion 280 kb upstream of the *FOXL2* gene is responsible for the polled intersex phenotype (PIS), causing ovary-to-testis sex reversal in XX homozygotes (Pailhoux et al., 2001). The PIS phenotype is associated with the loss of expression of *FOXL2* and three long non-coding RNAs (Pailhoux et al., 2001; Beysen et al., 2005). Generation of a goat *FOXL2* KO model demonstrated that loss of *FOXL2* expression alone is responsible for PIS and XX ovary-to-testis sex reversal phenotype (Boulanger et al., 2014). In both XX *PIS* mutant and XX *FOXL2* KO gonads, expression of pro-ovarian genes *RSPO1*, *RSPO2*, *WNT4*, *CYP19* and *FST* is downregulated while the expression of pro-testis genes *SOX9*, *AMH*, *DMRT1* and *CYP17* is upregulated (Figure 5C) (Boulanger et al., 2014; Elzaiat et al., 2014).

During goat ovarian differentiation, granulosa cells in the ovarian medulla co-express FOXL2 and CYP19A1, while RSPO1 is expressed in the ovarian cortex (Pannetier et al., 2006; Kocer et al., 2008). *In vitro* studies showed that FOXL2 could regulate *CYP19A1* promoter activity. *DMRT1* up-regulation in XX *PIS* mutant gonads precedes *SOX9* up-regulation, suggesting a critical role of DMRT1 for testis differentiation in goats (Figure 5C) (Elzaiat et al., 2014). *RUNX1* is highly expressed in XX gonads during sex differentiation, but its function in goat ovary remains to be determined (Nicol et al., 2019). Hence, ovarian differentiation in goats is mainly mediated by *FOXL2* and partially regulated by the RSPO1/WNT4 pathway. *FOXL2* prevents activation of the pro-testis program through antagonizing of *DMRT1* (Figure 5C).

#### Human

In humans, the identification of genes involved in granulosa cell differentiation and ovarian development derives from analyses of individuals presenting Differences of Sex Development (DSD) (Figure 5D), combined with knowledge acquired from genetically modified mouse models. DSDs are congenital conditions in which the development of chromosomal, gonadal, or anatomical sex is atypical. Analyses of *SRY*-negative cases of XX testicular DSD and XY feminization or dysgenesis, led to the identification of key pro-ovarian genes. Duplication of the X-linked gene *NR0B1* (also called *DAX1*) causes 46, XY male-to-female sex reversal with gonad dysgenesis (Bardoni et al., 1994; García-Acero et al., 2019). In the mouse, overexpression of *Nr0b1* in XY gonads inhibits NR5A1 activation of *Sox9* enhancer, resulting in ovotestis development (Ludbrook et al., 2012). Duplication of a genomic region containing both *WNT4* and *RSPO1* genes results in XY gonadal dysgenesis (Jordan et al., 2001). *WNT4* missense mutation causes embryonic lethal SERKAL syndrome with 46, XX (ovo)testicular DSD (Mandel et al., 2008). Homozygous mutations for *RSPO1* are associated with XX DSD and testicular or ovotesticular development (Parma et al., 2006; Tomaselli et al., 2011; Naasse et al., 2017; Tallapaka et al., 2018). These findings confirm the key role that *WNT4* and *RSPO1* play in granulosa cell differentiation and early ovarian differentiation in humans. Further analyses revealed that *RSPO1* is upregulated in the ovary between 6 and 9 wpc, whereas the expression of *WNT4* and *CTNNB1* is not significantly different between ovary and testis at this stage (Tomaselli et al., 2011).

In humans, contrary to other vertebrate species, no *FOXL2* mutations are linked to 46, XX (ovo)testicular DSD. Heterozygous mutations of *FOXL2* are associated with the autosomal dominant *Blepharophimosis Ptosis Epicanthus Inversus syndrome* (BPES), which causes early premature ovarian insufficiency in BPES type I (Crisponi et al., 2001). This raises the question whether *FOXL2* still acts in early granulosa cell differentiation/ovarian determination in humans, or if its role is limited to later granulosa cell function in folliculogenesis.

*NR5A1*, encoding for Steroidogenic Factor 1 (SF-1), is probably one of the most complex genes studied in DSDs, associated with a wide spectrum of DSD cases ranging from XY gonadal dysgenesis to male infertility, as well as primary ovarian insufficiency in women (Domenice et al., 2016). Multiple cases of 46, XX (ovo)testicular DSD were linked to a heterozygous missense mutation p.R92W in the DNA binding domain of *NR5A1* (Bashamboo et al., 2016; Baetens et al., 2017). Remarkably, some XX carriers were asymptomatic and fertile, whereas in one family, this same mutation also led to XY DSD in a sibling. It remains unclear how the phenotype of this point mutation is so variable. Generation of a mouse model carrying this mutation caused XY dysgenesis, but not XX sex-reversal, suggesting some differences in p.R92W mutated *NR5A1* activity between human and mouse (Miyado et al., 2016). This capacity to cause both XX and XY DSD is likely due to *NR5A1* involvement in early gonadogenesis and establishment of bipotential supporting cells (Hanley et al., 1999). Like *NR5A1*, the gene *WT1* is involved in bipotential gonad formation and mutations were previously only associated with XY DSD. However, several pathogenic variants of *WT1* have been associated with testis differentiation in XX individuals (Gomes et al., 2019; Eozenou et al., 2020; Sirokha et al., 2021; Ferrari et al., 2022). For both *NR5A1* and *WT1* mutations, it is suspected that these pathogenic variants impair or modify the capacity of NR5A1 or WT1 to physically interact with β-catenin, therefore compromising ovarian differentiation (Bashamboo et al., 2016; Eozenou et al., 2020).

Heterozygous mutation or genomic deletion of *NR2F2* (or *COUP-TFII*) cause testis development in XX individuals (Bashamboo et al., 2018; Carvalheira et al., 2019). These genetic defects were also associated with BPES, a syndrome usually associated with *FOXL2* mutations. NR2F2 protein is detected exclusively in interstitial cells of the human ovary at 9 wpc and not in FOXL2+ granulosa cells. It is unclear how *NR2F2* impacts granulosa cell differentiation and how its loss of function results in testis tissue development. *NR2F2* may act indirectly from the stroma compartment. Another possibility is that *NR2F2* is expressed early on in supporting cell precursors before becoming exclusively expressed in the interstitial cells, and its loss impacts supporting cell capacity to differentiate into granulosa cells. Again, the DSD cases of *NR2F2* mutations highlight the differences in early gonad differentiation between the mouse and human, as loss of *Nr2f2* in the mouse ovary is not associated with ovary-to-testis sex reversal (Zhao et al., 2017).

Many cases of *SRY*-negative XX ovotesticular DSD remain unsolved. Mutations may be located in non-coding genomic regions that control the expression of known pro-ovarian genes. For instance, rare single nucleotide variations in putative enhancers for *WNT4* and *RSPO1* were found in some patients with impaired ovarian development (Nakagawa et al., 2022). The functional significance of these putative enhancers and their mutations require additional experiments for confirmation.

Transcriptomic analyses of human fetal ovaries by microarray (Mamsen et al., 2017) and bulk RNA-seq (Lecluze et al., 2020) provided new insights into the dynamics of expression of the genes identified as involved in XX DSD. Some notable differences with the mouse model were identified: for instance, expression of *NR5A1* is maintained longer in the human fetal ovary during early sex-differentiation (Mamsen et al., 2017; Lecluze et al., 2020) whereas it is quickly decreased in the mouse fetal ovary at the onset of sex determination (Ikeda et al., 1994). This difference in *NR5A1* expression dynamics may explain the difference in phenotype with the *NR5A1* p.R92W missense mutation that results in XX testicular DSD in humans but not in the mouse (Miyado et al., 2016). Another discrepancy between mouse and human implicates *WNT4*: while *Wnt4* expression is sexually dimorphic during sex determination in the mouse, human *WNT4* expression is not significantly different between sexes, despite its involvement in 46, XX testicular DSD (Mamsen et al., 2017). On the other hand, *RSPO1* and *AMHR2, LGR5,* and *RUNX1* are among the earliest pro-ovarian genes expressed in human fetal ovaries with strong sexual dimorphism around 6 wpc/early 7 wpc (Lecluze et al., 2020). Meanwhile, *FOXL2* becomes enriched in the human fetal ovary after 7 wpc, a bit later than these genes (Lecluze et al., 2020). This may explain why mutations in *FOXL2* gene does not lead to ovary masculinization in humans but instead to a folliculogenesis phenotype.

### Evolutionary perspectives

Altogether, although some factors like the RSPO1/WNT/β-Catenin pathway and FOXL2 have a broadly conserved role in ovarian differentiation, some divergence led to clade-specific, and even species-specific sub-functionalization. Among the conserved pathways, the RSPO1/WNT/β-catenin signaling is a strong driver of ovarian differentiation from fish to human. In mammals, this pathway seems to have taken the front seat, as the strongest ovary-to-testis sex-reversal phenotype from a single gene deletion is caused by interrupting this pathway in mice. Similarly in humans, rare cases of XY DSD caused by a genomic duplication involve the locus encompassing *RSPO1* and *WNT4*. While *FOXL2* expression in the ovary is highly conserved throughout vertebrate species, its functions in either primary sex determination, ovarian maintenance or folliculogenesis has evolved within vertebrate clades, and even within species from the same clade. This phenomenon may be related to the evolution of *DMRT1* functions in testis determination and maintenance, as *FOXL2/DMRT1* directly antagonize each other. Another possibility is that *FOXL2* functions evolved with the role of estrogens in ovarian differentiation through *FOXL2’s* capacity to directly control *CYP19A1* expression and cooperate with estrogen receptors. Indeed, *FOXL2* seems to play a more prominent role in ovarian differentiation of species that rely on estrogen signaling. Tilapia is a good example as *foxl2* and *cyp19a1a* KO both lead to identical phenotypes. In most species, *FOXL2* and *CYP19A1* are expressed in the same cell populations, whether it is granulosa cells, or steroidogenic cells in fish ovaries, supporting the hypothesis that *FOXL2* directly controls *CYP19A1* expression. An exception to the rule is the rabbit, as *CYP19A1* and *FOXL2* are expressed in the cortex and medulla, respectively, in the fetal ovary. This suggests that *FOXL2* functions are decoupled from *CYP19A1* activation in the rabbit fetal ovary and raises the question of what molecular pathways control *CYP19A1* expression in the rabbit ovarian cortex. Therefore, it is still unclear how the mode of action of FOXL2 has evolved depending on the species.

Similar to *FOXL2*, the role of other genes in ovarian differentiation has evolved within the same clades. For instance, comparison of some human DSD with mouse models for an identical mutation led to surprisingly different phenotypes. These differences may be caused by difference in timing of morphogenesis between the species, changing the influence some genes like *NR5A1* or *NR2F2* can have on ovarian differentiation.

Finally, one clear evolutionary difference in vertebrate ovarian differentiation is the impact germ cells have on ovarian identity. While germ cell numbers influence the fate of gonadal somatic cells and the maintenance of granulosa cell identity in fish, it is not the case in mice (Maatouk et al., 2012). In fish, ovarian germ cells can also autonomously control their own fate through *foxl2l*, a gene closely related to *FOXL2*. During evolution, this gene was lost in terrestrial vertebrates, and germ cell fate became exclusively dependent on supporting cell fate. It is unclear whether the presence of this *foxl2l* gene also influences the impact of germ cells on supporting cell identity. While germ cells do not influence granulosa cell determination in mice, differentiation of *Lgr5*+ pre-granulosa cells into *Foxl2*+ granulosa cells is delayed in mutant ovaries lacking germ cell-specific genes *Nanos3* or *Figla* (Fukuda et al., 2021). Therefore, the key role of supporting/germ cell communication in ovarian determination has been lost during vertebrate evolution, but it remains critical for proper cortical granulosa cell differentiation in mice.

## Conclusion and perspectives

From fish species with an incredible range of sex determination systems and gonadal plasticity, to chicken with left-right asymmetrical ovarian development, to placental mammals whose gonad differentiation occurs in the environment of the womb and maternal hormones, evolution of ovarian differentiation reveals both relatively conserved and unique features within vertebrate clades and species. This demonstrates that evolution of ovarian differentiation is non-linear, and some unique evolutionary events occurred independently among species. Of course, this review on vertebrate ovarian differentiation does not intend to cover all vertebrate species, and other vertebrates such as amphibians, lizards or turtles have their own story to tell.

Intriguing new potential players in ovarian differentiation require further study in different vertebrate clades. For instance, while *RUNX1* plays complementary roles with *FOXL2* in maintaining fetal granulosa cell identity in mice, its ovarian function in other vertebrate species remains to be determined. The potential roles of *OSR1*, a gene enriched in avian pre-granulosa cells, and *NR2F2*, a gene linked to ovary-to-testis sex reversal in humans, need to be examined in other species. Beyond granulosa cells, the functions of other ovarian cell populations such as NR2F2+ interstitium or germ cells cannot be ignored. For instance, *Lhx2* gene in germ cells is involved in repression of endothelial cell migration in the mouse developing ovary (Singh et al., 2022).

The emerging single-cell sequencing technologies are becoming a game changer in the study of developmental processes, providing single-cell resolution of tissue composition and cell lineage differentiation trajectories. Single-cell RNA-seq methods have already been used to study gonad differentiation in various species (Estermann and Smith, 2020) and more studies come out regularly. In addition, the use of related techniques such as spatial transcriptomics and single-cell ATAC-seq will allow the building of a comprehensive developmental cell atlas of gonad differentiation and further improve the comparative analyses of granulosa cell origins and differentiation kinetics within vertebrate clades. For instance, the Human Gonad Development Atlas, a part of the Human Developmental Cell Atlas (HDCA), is aimed to create a comprehensive map of cells during human fetal development using 2D/3D imaging as well as single cell multiomics (Haniffa et al., 2021; Garcia-Alonso et al., 2022). Beyond gene expression, identifying non-coding regulatory elements, enhancers with potential granulosa cell signatures and cell-specific chromatin dynamics associated with different stages of ovarian differentiation will further improve our knowledge of ovarian differentiation and maintenance of its identity.

## Glossary

ADAMTS9: A disintegrin and metalloproteinase with thrombospondin motifs 9
AMH: Anti-Mullerian hormone
BMP15: Bone morphogenetic protein 15
BMPR2A/B: Bone Morphogenetic Protein Receptor Type 2 a/b
BPES: Blepharophimosis–ptosis–epicanthus inversus syndrome
COUPTFII: Chicken Ovalbumin Upstream Promoter Transcription Factor 2
CTNNB1: Catenin Beta 1
CYP17A1: Cytochrome P450 Family 17 Subfamily A Member 1
CYP19A1: Cytochrome P450, family 19, subfamily A, polypeptide 1, also called aromatase
CYP26B1: Cytochrome P450 Family 26 Subfamily B Member 1
DAX1: Dosage-sensitive sex reversal (DSS), Adrenal hypoplasia congenital critical region on the X chromosome, gene 1
DDX5: DEAD-Box Helicase 5
DKK1: Dickkopf WNT signaling pathway inhibitor 1
DMRT1: Doublesex and mab-3 related, transcription factor 1
Dmy: DM-domain gene on the Y chromosome
dpc: Days post coitum
dph: Day post hatching
DSD: Differences or disorders of sex development
E: Embryonic day
E2: Estradiol-17b
EMT: Epithelial–Mesenchymal Transition
EMX2: Empty Spiracles Homeobox 2
ERα/β: Estrogen receptor alpha/beta
ESR1/2: Estrogen receptor 1 / 2
FOXL2: Forkhead box L2
FST: Follistatin
GDF9: Growth differentiation factor 9
GSDF: Gonadal soma derived growth factor
IGF3: Insulin-like growth factor-3
JCM: Juxtacortical medulla
KO: Knockout
LGR4: Leucine-rich repeat-containing G-protein coupled receptor 4
LGR5: Leucine-rich repeat-containing G-protein coupled receptor 5
LHX9: LIM Homeobox 9
NOBOX: NOBOX oogenesis homeobox
NR2F2: Nuclear Receptor Subfamily 2 Group F Member 2 (*COUP-TFII*)
NR5A1: Nuclear receptor subfamily 5 Group A Member 1 (SF-1)
NR5A2: Nuclear receptor subfamily 5 Group A Member 2 (LRH-1)
NR0B1: Nuclear Receptor Subfamily 0 Group B Member 1 (DAX1)
OSR1: Odd-Skipped Related Transcription Factor 1
PAX2: Paired box gene 2
PGCs: Primordial Germ Cells
PIS: Polled Intersex Syndrome
PITX2: Paired Like Homeodomain 2
PRMT5: Protein arginine methyltransferase 5
RALDH2: retinaldehyde dehydrogenase 2 (ALDH1A2)
RBPMS2: RNA-binding protein of multiple splice forms 2
RSPO1: R-spondin 1
RUNX1: Runt-related transcription factor 1
scRNA-seq: single-cell RNA sequencing
SERKAL: Sex reversal with dysgenesis of kidneys, adrenals, and lungs
SF-1: Steroidogenic factor-1
SOX9: SRY-related HMG box 9
SRY: Sex-determining region on the Y chromosome
TALEN: Transcription activator-like effector nuclease
TGF β: Transforming growth factor beta
TGIF1: TGFB Induced Factor Homeobox 1
WNT4: Wingless-type MMTV integration site family, member 4
wpc: Weeks post coitum/conception
WT1: Wilms’ tumor suppressor 1
